# Climate change in Europe between 90 and 50 kyr BP and Neanderthal territorial habitability

**DOI:** 10.1371/journal.pone.0308690

**Published:** 2025-02-26

**Authors:** Anna Degioanni, Sandrine Cabut, Silvana Condemi, Robin S. Smith

**Affiliations:** 1 Aix-Marseille Universite, CNRS, Minist, Culture, LAMPEA, Aix-en-Provence, France; 2 Aix-Marseille Universite, EFS, CNRS, ADES, Marseille, France; 3 Department of Meteorology, NCAS, University of Reading, Reading, United Kingdom; Universita degli Studi di Ferrara, ITALY

## Abstract

After having lived as the dominant human species in Europe for over 200 kyr, *Homo neanderthalensis* (the Neanderthals) disappeared around 40 kyr BP (Before Present) Higham T (2014). Competition with *Homo sapiens*, who arrived in Europe around the same time, is often invoked to explain this extinction. Others have argued that climate change may have reduced the living space of this population making its disappearance more rapid. In order to test the climate change hypothesis we modelled the Neanderthals’ ecological niches in Europe between 90 and 50 kyr BP through paleoenvironmental reconstructions and Eco-Cultural Niche Modelling. We selected five environmental variables (orographic height, mean annual precipitation, mean temperature of the coldest month, carrying capacity and friction, see below) from climate model simulations of 5 periods between 90 and 50 kyr BP in Europe. We used Structural Similarity (SSIM) index to compare the probability maps of suitable niches to Neanderthals performed by Maxent. After a strong initial environmental change between the first (P1 = 90 to 83 kyr BP) and second (P2 = 83 to 69 kyr BP) periods, our results show that large areas highly suitable for Neanderthal occupation persisted across Europe. As our results show an increase/stability of the areas suitable to Neanderthals, the question of the cause of the decrease or displacement of the Neanderthal population towards southern Europe after this climatic change remains open.

## Introduction

Neanderthals evolved in Europe over a long period. The setup of their features can be followed through the MIS (marine oxygen-isotope stage) 7 and 6. They dominated unchallenged until MIS 3, when *Homo sapiens* gradually settled in Europe [1]. Around 40 kyr BP, Neanderthals were totally replaced by *Homo sapiens* [2].

Neanderthals were well-suited to Europe [3], remaining in this region through a succession of major glacial periods characterized by extensive ice sheets and warmer interglacials. They lived in heterogeneous environments extending from temperate grasslands to the tundra biome, indicating a high degree of plasticity in relation to the environment [4].

A plethora of studies (but almost all on *Homo sapiens)* investigate the effect of climate on human dispersal and evolution [5].

In this article we focus on the period between 90 to 50 kyr BP. Throughout this period, temperatures and precipitation fluctuated between cold and dry stadials and warm and wet interstadials. The climate, which was significantly colder than today, led to the formation of ice caps and glaciers responsible for a fluctuation of sea levels between 40 to 80 meters lower than today [6–9]. This drop in sea level led to the unveiling of land bridges and thus allowed humans to colonize lands that were previously inaccessible on foot, for example between the Italian and the Balkan peninsulas. The presence of extensive ice caps also had an impact on precipitation, which was lower than today. Because of the low precipitation and temperatures, the environment consisted mostly of open grassland, tundra and steppe in Europe and it was semi-arid in the Middle East [10].

During this period, the presence of Neanderthals is attested by numerous sites that have yielded fauna, lithic assemblages and sometimes, Neanderthal remains and it is therefore possible to establish the spatial distribution of this population. Nevertheless, taphonomic factors and the scarcity of excavation in certain areas greatly limits our knowledge of the full extent of the Neanderthal presence. However, it is possible to characterize the environments of the sites that have been investigated and thus identify other areas where Neanderthal presence may have been suitable. We use this approach to investigate the ecological niches that represent the range of environmental conditions in which the Neanderthals could have survived.

It is our intention to examine whether there was a reduction in the areas suitable for Neanderthal occupation in Europe between 90 to 50 kyr BP, we are therefore interested in the period before the arrival of the *Homo sapiens*.

To address this we divide the timespan 90–50 kyr BP into subperiods, reconstruct the ecological niches for each period and examine whether there was a spatial change of these niches over time.

Above all, we are interested in quantifying the full extent of the suitable space theoretically available to Neanderthals at the time of their demographic decline.

## Material and methods

**Fig 1:**
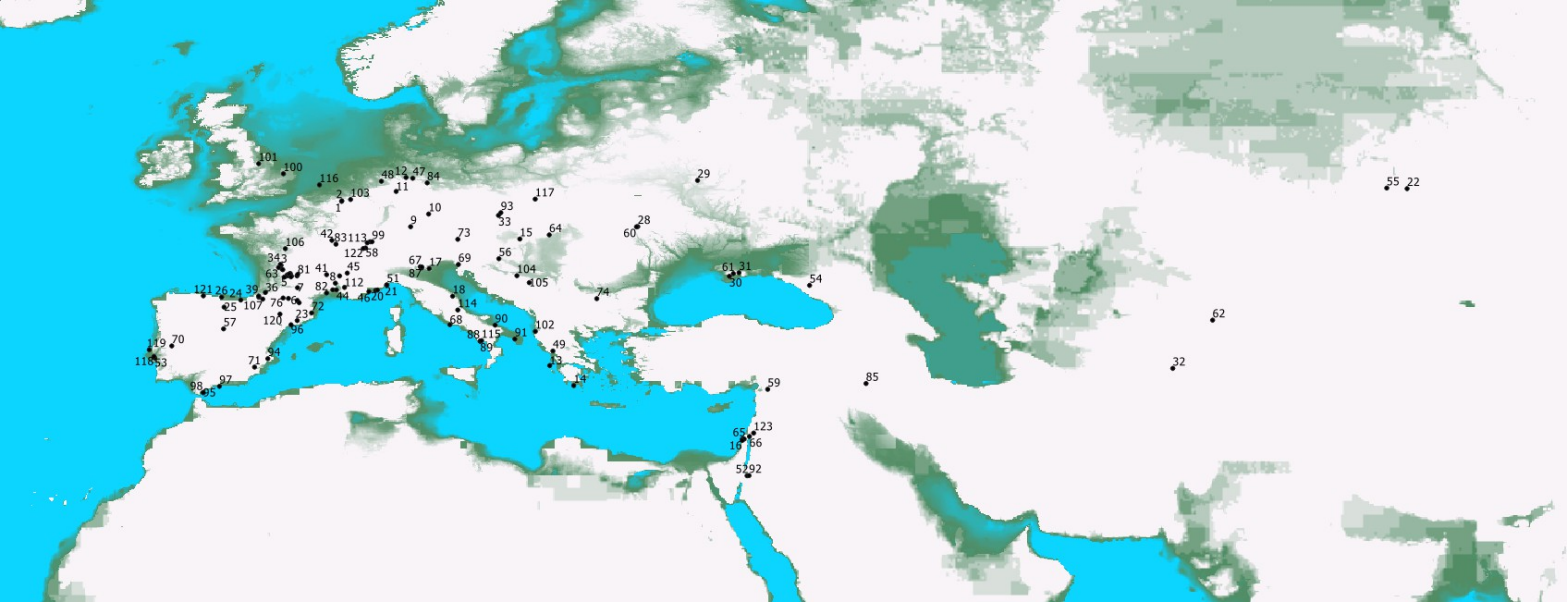
Location of the 123 Neanderthal fossil sites (*1 Goyet Caves; 2 Scladina; 3 La Chaise Bourgois-Delaunay; 4 La Ferrassie; 5 Roc de Marsal; 6 Le Portel; 7 La Rouquette; 8 Grotte de Saint Marcel; 9 Hohlenstein-Stadel; 10 Hunas; 11 Buhlen; 12 Sarstedt; 13 Kefalonia; 14 Grotte de Kalamakia; 15 Tata; 16 Tabun; 17 Grotta Maggiore di S*. *Bernardino; 18 Grotta de’ Santi; 19 Riparo Mochi; 20 Grotta Madonna dell’Arma; 21 Arma delle Manie; 22 Altai Mountain; 23 Abric Romani; 24 Grotte de Lezetxiki; 25 Valdegoba; 26 El Castillo; 27 Alle-Noir Bois; 28 Korolevo; 29 Staroselye; 30 Kabazi; 31 Prolom II; 32 Teshik-Tash; 33 Kulna; 34 Marillac; 35 Sandougne et abri Brouillard; 36 Grotte de L’Hyene; 37 Regourdou; 38 Grotte d’Unikoté; 39 Grottes d’Isturitz et d’Oxocelhaya; 40 Pech l’Azé1; 41 Abri Laborde; 42 La Brèche de Genay; 43 Grotte de la Verrerie; 44 Loton; 45 Préletang; 46 Abri Pié Lombard; 47 Salzgitter-Lebenstedt; 48 Warendorf; 49 Kokkinopilos; 50 Riparo Zampieri; 51 Caverna delle Fate; 52 Tor Faraj; 53 Conceicao; 54 Mezmaiskaya Cave; 55 Chagyrskaya; 56 Divje Babe; 57 Pinilla del Valle; 58 Grotte de Cotencher; 59 Dederiyeh; 60 Molodova; 61 Kiik-Koba; 62 Obi-Rakhamat Grotto; 63 La Quina; 64 Subalyuk; 65 Ein Qashish; 66 Amud; 67 Grotta della Ghiacciaia; 68 Guattari; 69 Grotta di Cotariova; 70 Vilas ruivas; 71 Cueva Anton; 72 Banyoles; 73 Salzofen Cave; 74 Temnata Cave; 75 Fonseigner; 76 Grottes du Coupe-Gorge; 77 Sites du Cotentin; 78 Mauran; 79 Le Moustier; 80 Fieux; 81 La Chapelle aux Saints; 82 Hortus; 83 Grotte Boccard; 84 Königsaue; 85 Shanidar; 86 Kebara; 87 Riparo Tagliente; 88 Grotta Taddeo; 89 Grotta Tina; 90 Bisceglie; 91 Grotta di Uluzzo; 92 Tor Sabiha; 93 Ochoz; 94 El Salt; 95 Gibraltar 2; 96 Cova del Gegant; 97 Cueva del Boquette; 98 Gibraltar 1; 99 Région de la Löwenbourg; 100 Lynford Quarry; 101 Pin Hole Cave; 102 Kryegjata B; 103 Fonds de forêt; 104 Zobiste; 105 Kadar; 106 Roche-Cotard Cave; 107 Grotte de Gatzarria; 108 La Roquette; 109 Combe Grenal; 110 Grottes du Tuteil et de Caougno; 111 Moula Guercy; 112 Auzières 2; 113 Grotte Vaufrey; 114 Calascio; 115 Molare; 116 Northsea shore; 117 Stajnia; 118 San Antao do Tojal; 119 Furninha; 120 Gabasa; 121 El Sidron; 122 Grotte des Plaints; 123 Quneitra)*. Topographic data ‘NOAA National Centers for Environmental Information. 2022: ETOPO 2022 15 Arc-Second Global Relief Model. NOAA National Centers for Environmental Information. https://doi.org/10.25921/fd45-gt74. Accessed 2024".

To more clearly characterise the stages of environmental change in the area we are studying we divide our study period into 5 subperiods between 90 and 50 kyr BP, each with its own reasonably uniform characteristics in the biome reconstructions of Hoogakker [11]: P1 = 90 to 83 kyr, P2 = 83 to 69 kyr, P3 = 69 to 64 kyr P4 = 64 to 51 kyr and finally P5 = 51 to 50 kyr. We focus our analysis on Eurasian territory between 20°W-100°E and 20°N-65°N, compiling data from 123 Neanderthal fossil sites classified by georeferenced occurrence location and subperiod (Fig 1, map made using [12]). We use the date of the layer as indicated in the reference publication (see S1 File).

### Paleo environmental data

To characterize the relevant environmental properties of the territory we select five variables: orographic height, mean annual precipitation, mean temperature of the coldest month, carrying capacity and friction.

Values for these environmental variables for our periods are derived from climate model simulations of the last glacial cycle [13,14], and complementary reconstructions of the ecological biomes implied by these climate simulations. Our biome reconstructions follow the method of Hoogakker [11], where the impact of known biases in the climate simulations is minimised by treating the simulations for each period as anomalies with respect to each model’s modern-day climate; the climate forcing used for our biome reconstructions is obtained by combining the anomalies for each period with modern observational datasets. See [11] for a full discussion of the treatment of the climate data and the method of biome reconstruction (see also S2 File). Although the climate model simulations we use are from an earlier generation of climate modelling, there is not a significant body of more recent work covering the time period we are studying (for instance the widely used protocol Paleo Model Intercomparison Project (PMIP) [15] do not include periods of glacial inception). In fact, a recent effort to make more widely available paleoclimate reconstructions [16], which relies on the dataset described by [17] for this period is itself derived from one of the same sets of climate simulations [13] as we consider here and uses a similar method of ecological reconstruction to ours. However, since our conclusions regarding the suitability of the environment for Neanderthal occupation are independent of which of our two climate reconstructions we use, for simplicity the ecological niche analysis presented in this paper uses reconstructions based on only one of the two climate models, the single, continuous transient simulation of [14].

Friction and carrying capacity are derived from the biome distribution reconstructed for the climate in each period. Friction is defined as the difficulty of moving in a given environment, with the density of vegetation affecting human movement. Values of friction have been considered, one for each global biome type (table 3.2 by [18]): ranging from 0.1 for environments where mobility is very easy, to 1 for environments across which it is practically impossible to spread, like ice sheets (see also S3 File). Carrying capacity is an indicator for the available quantity of flora and fauna, corresponding to the maximum number of individuals who can be supported in a given area within natural resource limits. Carrying capacity values were attributed to each biome type using the correspondence table 3.2 published by [18] (see also S3 File).

The anomaly method we use to obtain climate and biome reconstructions allows us to conduct all our analysis on the same 0.5° x 0.5° grid as the modern observational and topographic datasets we use in producing our environmental variables. To account for variations in sea level, and thus the area of land available at the coast, coastlines were adjusted by applying approximate global mean sea level changes characteristic for each period, and were created by extrapolating beyond the coastline with the average value of the nearest 3 existing inland grid points, estimated from [6]. Where these lowered coastlines did not exactly match those used in the climate model simulations, climate variables were extrapolated to the coast using the nearest 3 valid grid cells and these extrapolations used in the biome reconstruction.

### Maxent

The nature of our data, i.e. the presence of certain fossils, requires us to use a presence-only model to reconstruct the Neanderthals’ ecological niches. In fact, archaeological excavations inform us about where Neanderthal was present (presence data), but we have no certainty about where it was absent (absence data) (in contrast to, for example, systematic biological surveys that produce presence-absence data).

We chose to use Maximum Entropy Modelling (Maxent), specifically Maxent v3.4.4 [19]. The aim of Maxent [20,21] is to estimate a target probability distribution by finding the probability distribution of maximum entropy (i.e., that is most spread out, or closest to uniform), subject to a set of constraints (expressed in terms of simple functions of the environmental variables, called features; as for the predictors in usual modelling) that represent our incomplete information about the target distribution. This method has proven its effectiveness:

1. in presence-only data, indeed it really requires only presence data, and not pseudo-absence (i.e. absence data are locations randomly generated in the background, that’s to say sampling universe where environmental predictors are informed). This suppress the problem of “contaminated controls” [22,23], in which background data is treated as absence data, even though it is contaminated with presences;
2. in avoiding over-fitting by using l1-regularization [19];
3. with small sample sizes [24,25].

Given our small number of fossil sites and to avoid overfitting our data with an overly complex model, only linear, quadratic and hinge (like linear, but it is constant below a threshold) features were used to model the constraint link between the environmental variables and presence of Neanderthal. As the prevalence (proportion of occupied sites) of Neanderthals is typically unknown, Maxent’s default prevalence was not modified (value set by the software designers: Prevalence = 0.5). Likewise, for regularization and background settings, defaults (values set by the software designers: Regularization multiplier = 1; Maximum number of background points = 10,000, randomly sampled from covariate grids) were used. Results are shown in the cloglog output format, an estimate of the probability that the Neanderthals would have been able to live at a location, given the environmental variables at this location [26], which is classically called suitability in Species Distribution Modelling (SDM). Sites with small cloglog values are predicted to be unsuitable or only marginally suitable for the species. The constructed maps reflect these estimated probabilities, from red indicating high probability of suitable conditions for the Neanderthals, to blue indicating a low one.

In order to reduce the bias of model estimators and to validate the model by verifying the stability of its predictions, we implemented a bootstrap with 1000 replicates, each replicated sample obtained by sampling with replacement from the presence points, with the number of samples equaling the total number of presence points. Bootstrapping also allows us to compute the average Area Under the ROC (Receiver Operating Characteristic) Curve (AUC) and its standard deviation across models. AUC, applicable to any species modelling method, measures the quality of ranking of sites [27]. In the case of presence-only data, it can be interpreted as the probability that a randomly chosen presence site is ranked above a random background site [24]. Used as an indicator of good predictivity, models with values above 0.85 are considered potentially useful [28].

To evaluate the contribution of each environmental variable to the model, we used the jackknife test performed by Maxent. It gives the importance of each environmental variable computing the regularized gain (measure related to deviance) by training with each environmental variable first omitted, then used in isolation.

### Spatial comparison

In order to compare the probability maps obtained and to study the evolution in suitability for Neanderthal occupation of areas in Europe during the 5 periods, we used a Structural Similarity (SSIM) index [29]. This index compares continuous-valued spatial data and allows us to simultaneously consider the local magnitude and spatial structure. SSIM is bounded by (-1, 1), -1 indicating complete dissimilarity between the spatial structure of the compared maps, and 1 indicating that maps are identical. Due to the range and meaning of SSIM, we chose the arbitrary threshold of 0.2. Below this value, we consider the maps of the periods being compared to be dissimilar. This analysis was conducted using R-4.2.1 software [30].

## Results

### ROC and AUC

To assess the accuracy of our models we perform a ROC analysis (Curves and AUC Values) (S File) The ROC curves are similar for the five periods and AUC values are always bigger than 0.92 showing a high level of confidence in the model predictions (see S4 File).

For each period, data concerning temperature is always the environmental variable which contributes the most to the Maxent model (see S5 File). In each period the temperature is the most effective variable for predicting the suitability of each site for Neanderthal occupation: temperature appears to have the most useful information by itself and it is also the variable that decreases the gain the most when it is omitted. Precipitation is the other important variable while friction and carrying capacity bring little additional information. The environmental variables chosen are not orthogonal: temperature and precipitation are themselves strong controls on the biome reconstruction and thus the friction and carrying capacity terms we use [18].

### Maxent maps

**Fig 2:**
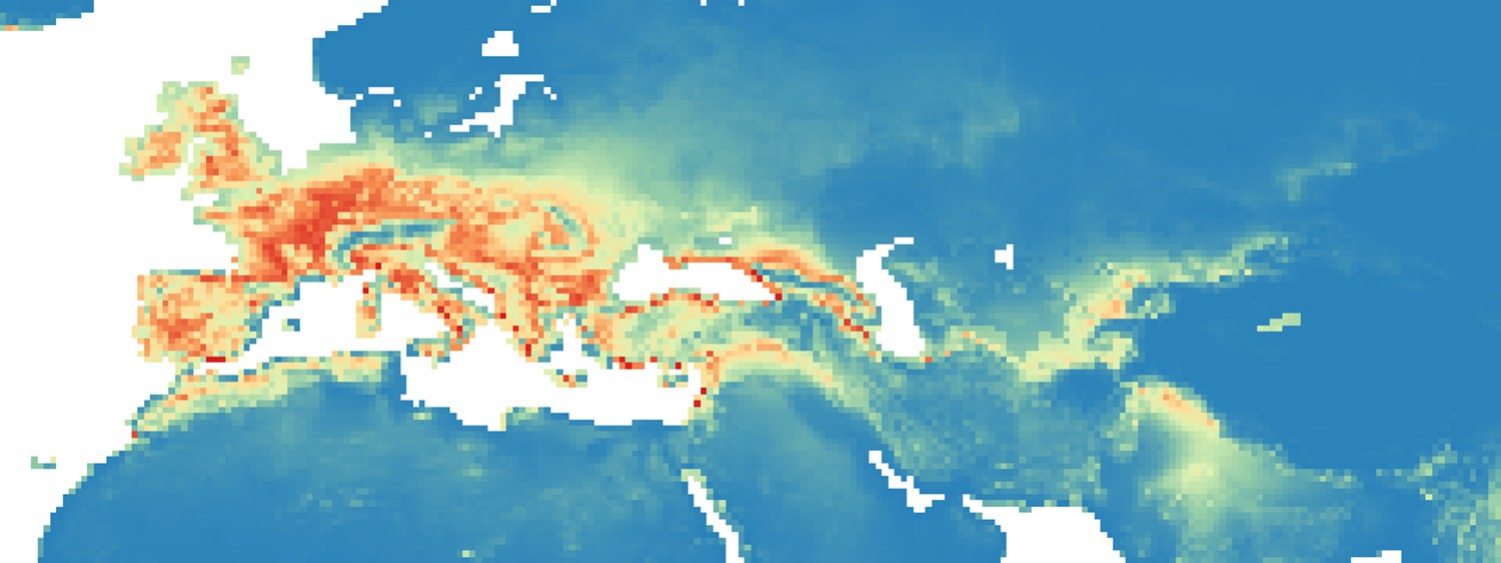
Heat maps (blue represent lower values (0–0.19), gradually transitioning (range of 0.20) to red, that is the higher values (0.81–1)) of Maxent modelling results: Estimated probability of suitable conditions for the Neanderthals, for period: P1 (90 kyr– 83 kyr).

**Fig 3:**
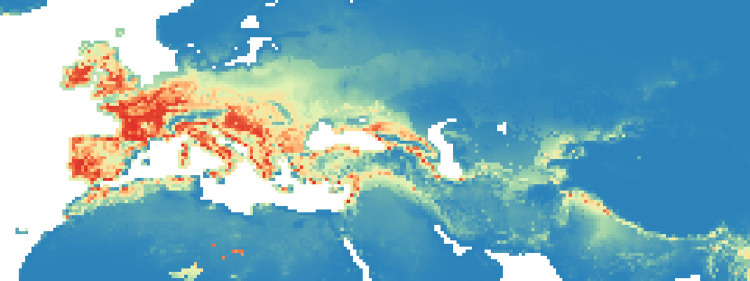
Heat maps of Maxent modelling results: Estimated probability of suitable conditions for the Neanderthal, for period P2 (83 kyr– 69 kyr).

**Fig 4:**
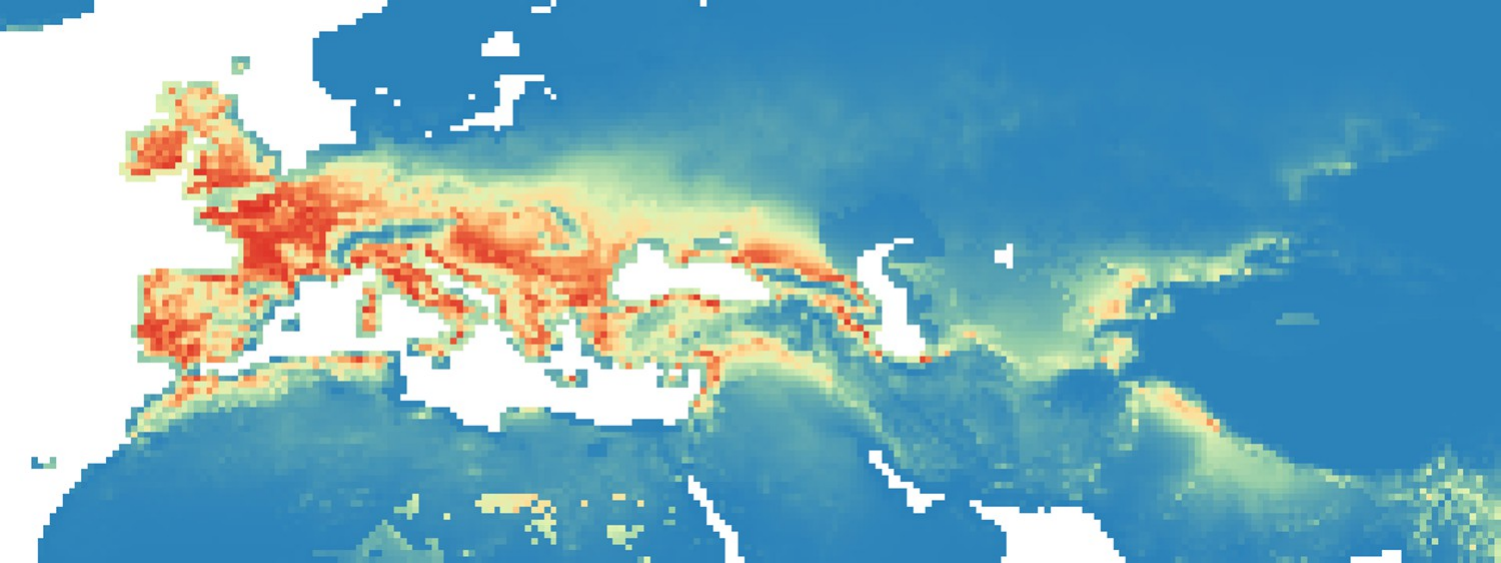
Heat maps of Maxent modelling results: Estimated probability of suitable conditions for the Neanderthals, for period P3 (69 kyr– 64 kyr).

**Fig 5:**
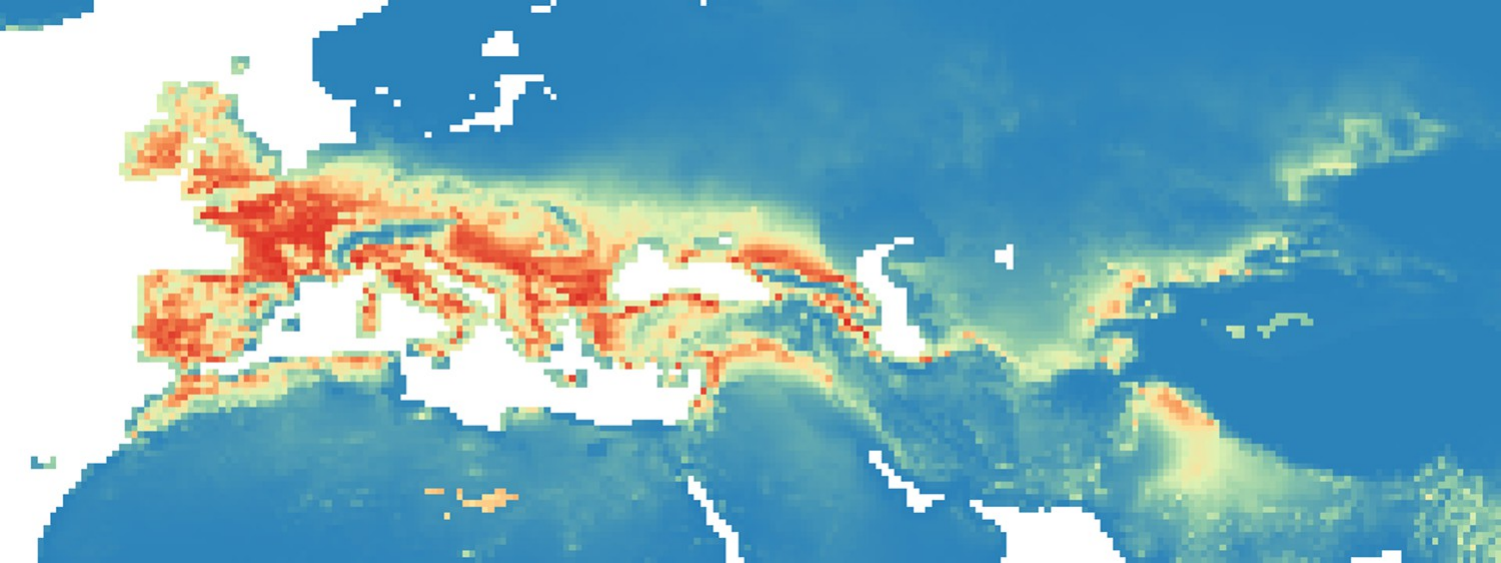
Heat maps of Maxent modelling results: Estimated probability of suitable conditions for the Neanderthals, for period P4 (64 kyr– 51 kyr).

**Fig 6:**
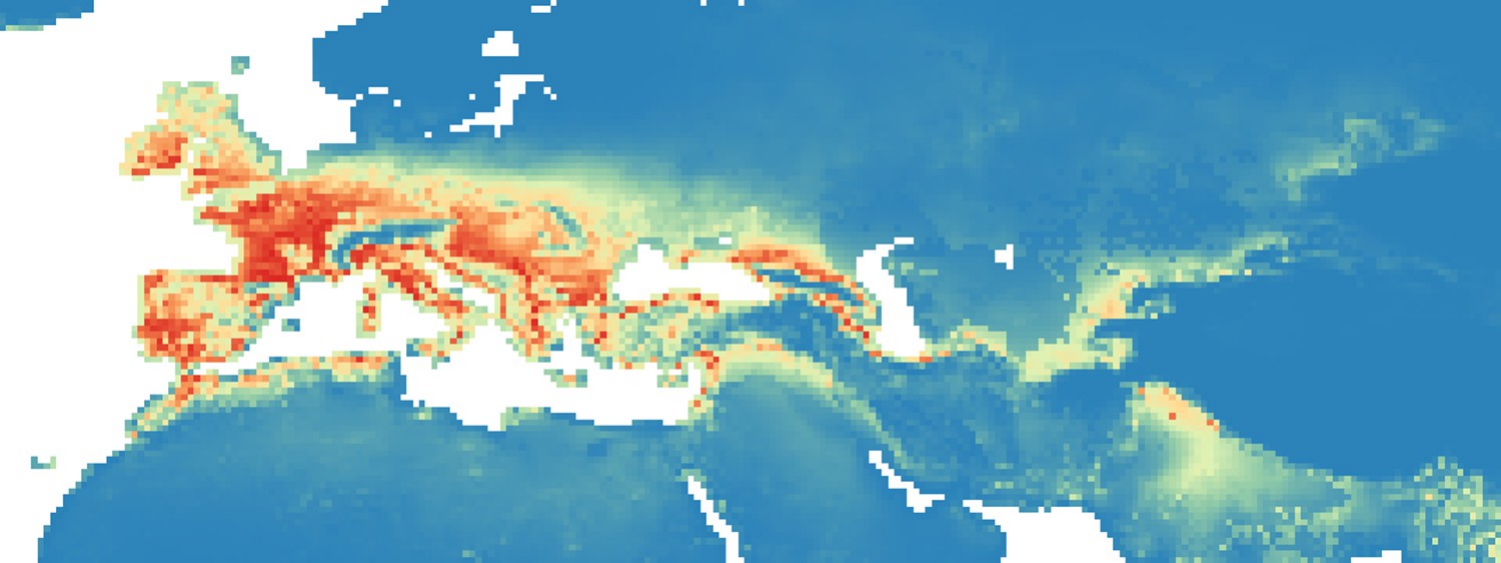
Heat maps of Maxent modelling results: Estimated probability of suitable conditions for the Neanderthals, for period P5 (51 kyr– 50 kyr).

Figs 2–6 show the results of the 1000 bootstraps carried out for each period on the output files concerning the estimation of probability of environmental suitability for Neanderthals. There are clearly many similarities among the maps representing the five successive periods extending from 90 to 50 kyr BP.

The five heat maps identify regions over time that had a very low (less than 0.2) or even zero probability of providing suitable ecological niches for Neanderthals. These areas are the Scandinavian regions, a large part of Eastern Europe, the Alps and part of the Mediterranean coast of the Iberian Peninsula. Other regions undergo changes in probability over time. A more detailed analysis of the characteristic habitability areas shown in these maps is presented in the following section.

The northern limit of habitability is not a pure function of latitude–Scandinavian regions have lower scores than Britain at all periods, while southern Europe seems to have been less suitable during early periods and more suitable later.

Over time, the percentage of ecological niches suitable to Neanderthals increases in P2 and P4 if we use a probability threshold greater than or equal to 0.8. If we refer to a more stringent probability *P* (greater than or equal to 0.9), then we obtain a constant increase in P2, a clear decrease in P3 and almost constant values thereafter (see S6 File).

**Fig 7:**
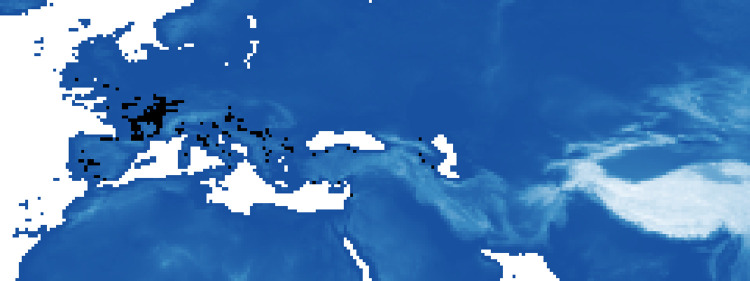
Highly favorable areas for the Neanderthals: Maxent’s estimated probability of suitable conditions allways greater than 0.8 over time (in black regions).

**Fig 8:**
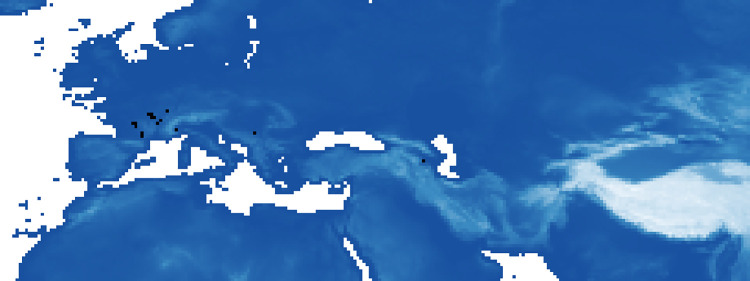
Highly favorable areas for the Neanderthals: Maxent’s estimated probability of suitable conditions allways greater than 0.9 over time (in black regions).

We also identify areas where, over time (between P1 and P5), the probability of suitable ecological niches has always been very high (0.9% of pixels always present over the periods with a probability greater than 0.8, Fig 7; and 0.1% of pixels with a probability greater than 0.9, Fig 8).

### Description of each period

#### Period 1 (90–83 kyr BP)

**Fig 9:**
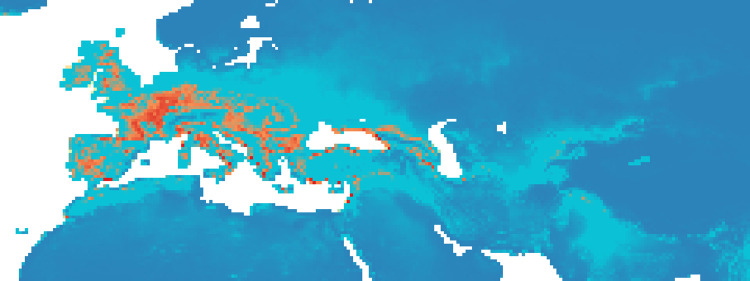
Estimated probability (Maxent’s cloglog outputs) of suitable conditions greater than 0.8 (orange to red), for period: P1 (90 kyr– 83 kyr).

In the first period, there was a higher probability of niches suitable for Neanderthals in the British Isles than for the other periods (Fig 2, the AUC value is 0.940): several pixels show values greater than 0.8 (Fig 9). The North and the West of the Iberian Peninsula, the Italian peninsula, the French and the German regions and the Balkans seem to have been very suitable regions and some suitable spots were present also in the Middle East and around and between Black and Caspian sea.

2.2% of the total emerged study area seem to have been very suitable for Neanderthals (*P* > 0.8), a more stringent probability (*P* > 0.9) suggests 0.4% of niches.

#### Period 2 (83–69 kyr BP)

**Fig 10:**
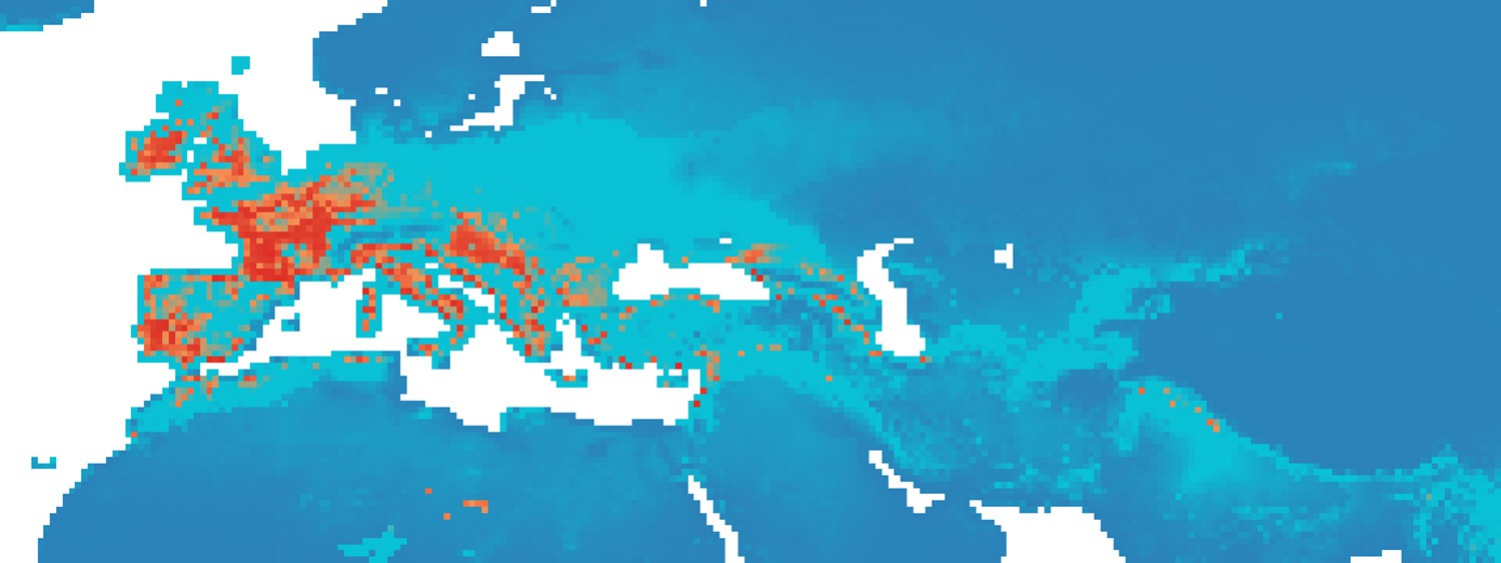
Estimated probability (Maxent’s cloglog outputs) of suitable conditions greater than 0.8 (orange to red), for period P2 (83 kyr– 69 kyr).

In the second period, the probability of suitable niches in the British Isles decreased compared to P1, but increased in Ireland and Iberia, Western Europe, the Italian peninsula and the Balkans (Fig 3). Our analysis shows an increase of probability in the east, around the Black Sea and in the Middle East (Fig 10). In this period, the percentage of suitable niches for Neanderthals increased and reached high values: 3.3% (*P* >0.8), and 1.6% for *P* > 0.9.

#### Period 3 (69–64 kyr BP)

**Fig 11:**
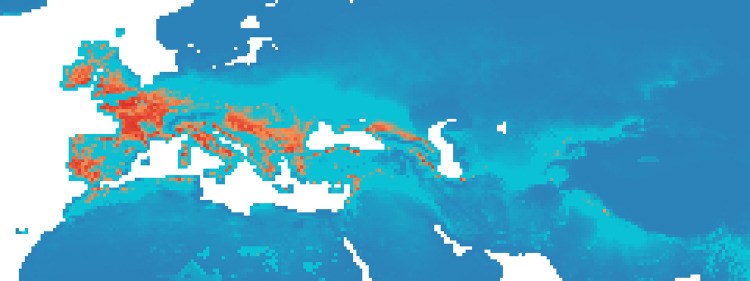
Estimated probability (Maxent’s cloglog outputs) of suitable conditions greater than 0.8 (orange to red), for period P3 (69 kyr– 64 kyr).

During the third period there was an increase in high probability niches in southern Europe, particularly in the western Iberian Peninsula, while its center and eastern side have very low values (Fig 4). The Italian Peninsula, the Tyrrhenian coast of the Balkans region and especially the Danube plain have high values during this period (Fig 11). In addition, the Anatolian Peninsula and Caucasian region seem to have been slightly more suitable, as was the Middle East. While the total area of suitable niches with a probability greater than 0.8 slightly decreased (only 3.2%), niches with the highest probability decreased (1%) compared to the earlier periods.

#### Period 4 (64–51 kyr BP)

**Fig 12:**
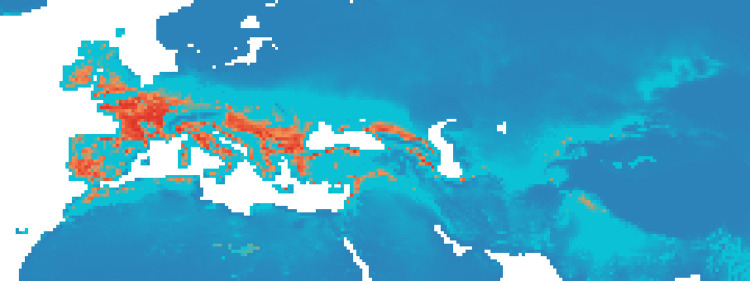
Estimated probability (Maxent’s cloglog outputs) of suitable conditions greater than 0.8 (orange to red), for period P4 (64 kyr– 51 kyr).

In the fourth period, there was an increase in high probability niches everywhere particularly in Balkans and Western Europe (Fig 5). In this period, the percentage of suitable niches for Neanderthals increases slightly both if we consider *P* > 0.8, and for *P* > 0.9 reaching respectively 3.5% and 1.1% (Fig 12).

#### Period 5 (51–50 kyr BP)

**Fig 13:**
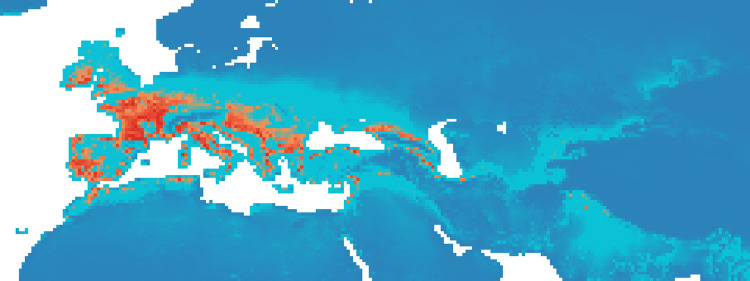
Estimated probability (Maxent’s cloglog outputs) of suitable conditions greater than 0.8 (orange to red), for period P5 (51 kyr– 50 kyr).

In this last period, there was a general decrease in probability niches, particularly in the Iberian and Balkan region, but an important and constant presence in Central Europe (Fig 6). The percentage of niches suitable for Neanderthal occupation (Fig 13) decreases slightly if we consider *P* > 0.8 (only 3.1%), and stays stable (1.1%) for *P* > 0.9.

### Evolution of niche suitability: Comparison of the similarity of periods

Examining the results of pairwise comparisons of Maxent probability maps for successive periods via SSIM allows us to understand the differences in geographical distribution that have been highlighted.

#### P1 vs P2

**Fig 14:**
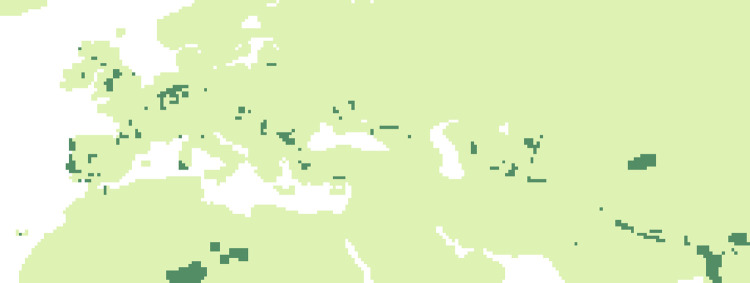
Comparisons by SSIM index of Maxent estimated probability maps for P1 vs P2. Dark green areas (less than 0.2) are considered as dissimilar.

The SSIM figure (Fig 14) shows areas where the dissimilarity between the two periods (90 kyr- 83 kyr and 83 kyr -69 kyr) are most important (using a limit of 0.2): these are several small areas scattered throughout the territory. In particular in the Iberian region, in France, and in the Danube plain.

#### P2 vs P3

**Fig 15:**
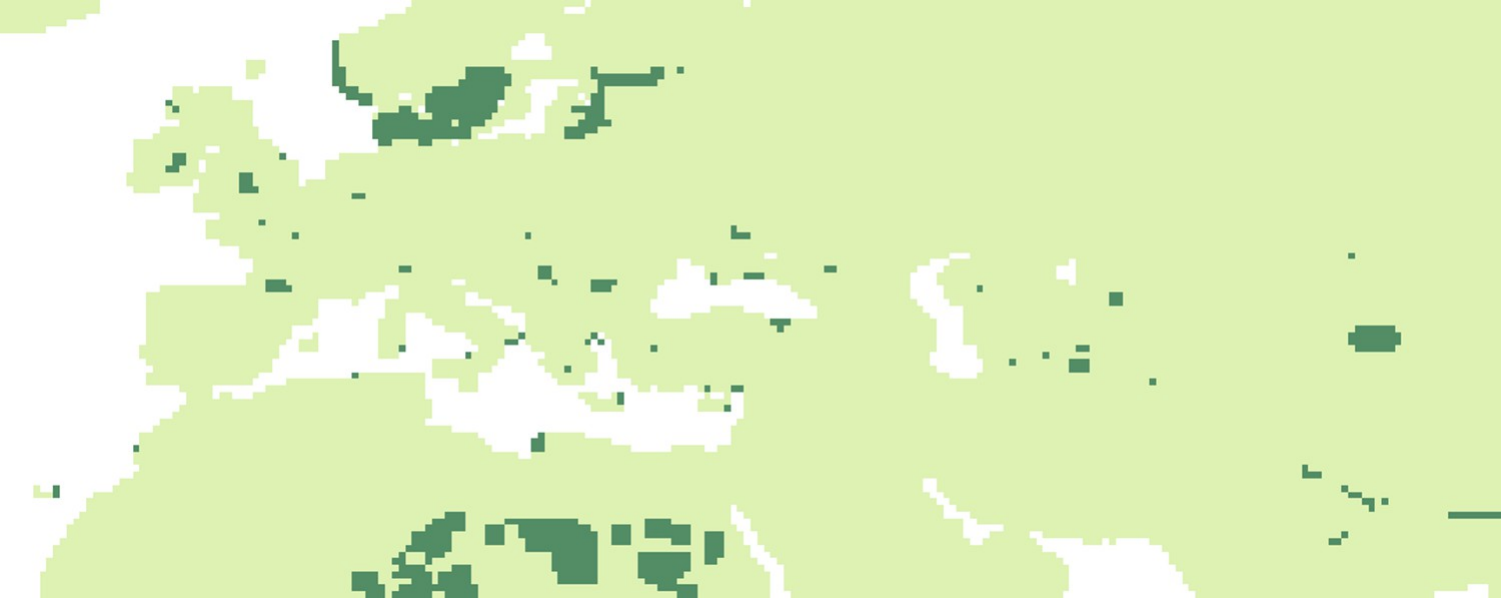
Comparisons by SSIM index of Maxent estimated probability maps for P2 vs P3. Dark green areas (less than 0.2) are considered as dissimilar.

Dissimilarities between the two periods (83 kyr -69 kyr and 69 kyr -65 kyr) (Fig 15) are particularly important (using a limit of 0.2) in the Scandinavian region and scattered across the rest of continental Europe.

#### P3 vs P4

**Fig 16:**
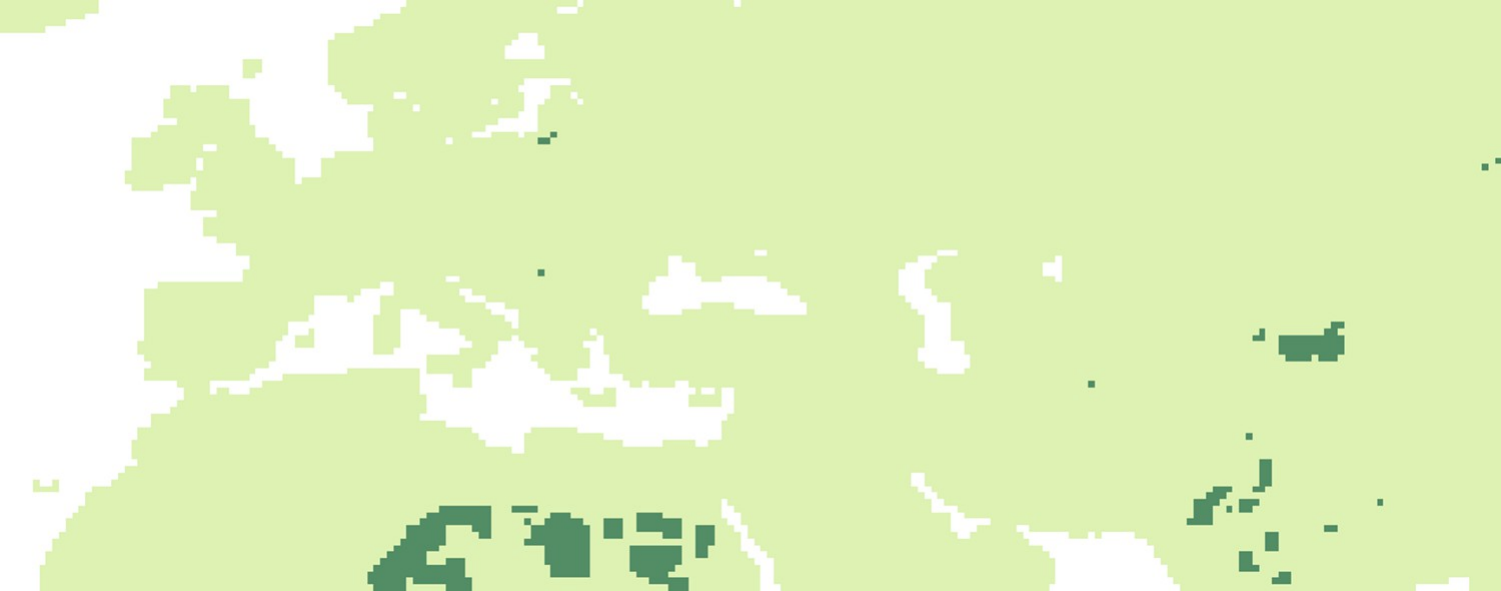
Comparisons by SSIM index of Maxent estimated probability maps for P3 vs P4. Dark green areas (less than 0.2) are considered as dissimilar.

Dissimilarities between the two periods (69 kyr -65 kyr and 65 kyr -51 kyr) (Fig 16) are very limited, only seen in 4 pixels in continental Europe.

#### P4 vs P5

**Fig 17:**
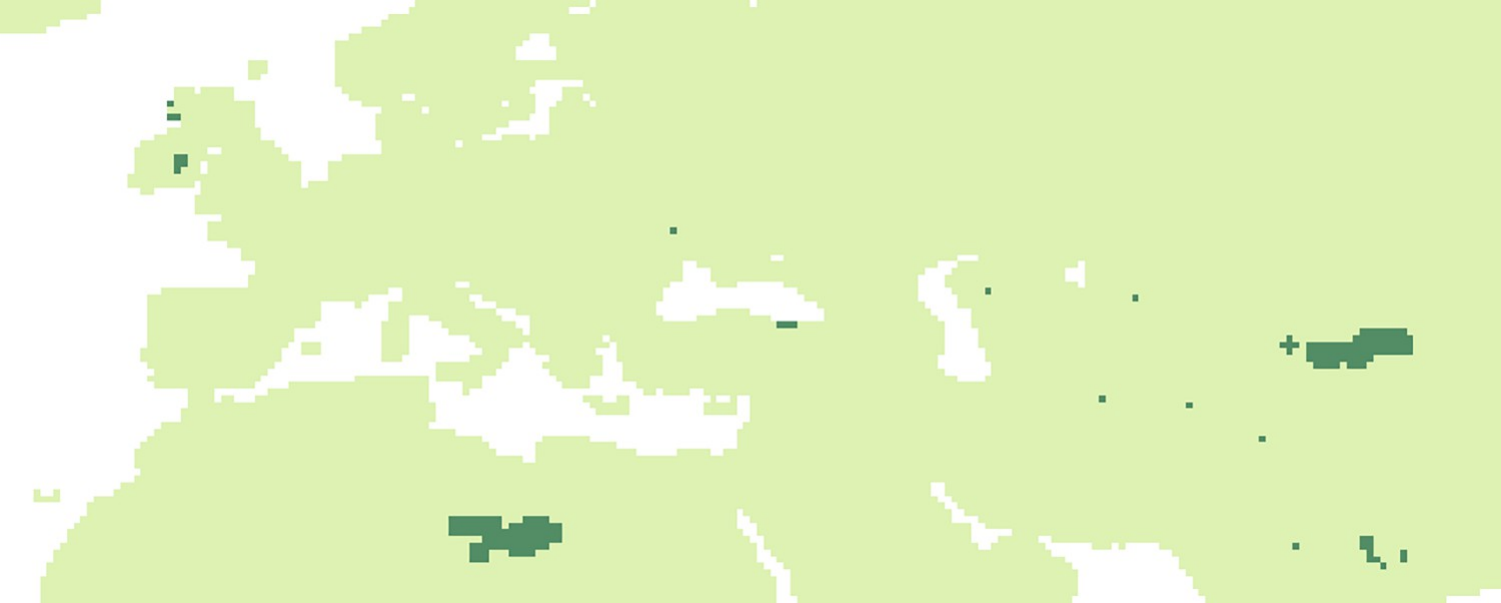
Comparisons by SSIM index of Maxent estimated probability maps for P4vs P5. Dark green areas (less than 0.2) are considered as dissimilar.

Dissimilarities between the two periods (65 kyr -51 kyr and 51 kyr -50 kyr) (Fig 17) are very small and concern the British region and isolated points in the rest of the territory.

## Discussion

One of the main flaws in prehistoric work is the sampling bias of sites (e.g. where Neanderthals may have been present). Indeed, our compiled georeferenced occurrence locations are taken from the literature, not from a planned sampling scheme. In order to overcome problem of possible biases on the studied sites and ensure the stability of our model, we implemented a bootstrap with 1000 replicates, each replicated sample obtained by sampling with replacement from the presence points, with the number of samples equaling the total number of presence points. Thus, it is difficult to differentiate whether presence in particular environments is because those locations were really preferable to Neanderthals or if this is due to the fact that a location has been the subject of the largest search efforts, has better conditions for conservation of fossils or simply because it is accessible for excavations. This question is one of the biggest sources of bias in the field of prehistory in particular, and archaeology in general. Our approach, presence-only SDM based on maximum entropy, helps to solve this problem and provide new indications of the probability of finding Neanderthal sites (with fossils, lithic industry or fauna) for the period 90 kyr—50 kyr in the European area.

It should be noted that our results encounter two biases. Firstly, our research only highlights areas that show environmental characteristics comparable to those of previously discovered Neanderthal sites. This is nevertheless a step forward, encouraging excavation of those areas with a high probability of discovering Neanderthal remains because we have identified areas with environmental characteristics strongly suitable for Neanderthal occupation. The second bias concerns the geographical scale of our study, which covers the entire European region and does not focus on the detail of more geographically limited areas. Indeed, it would also be preferable if the climate and ecological reconstructions our work relies on were explicitly conducted at higher spatial resolutions and able to more explicitly take account of very local conditions. However, our work nevertheless provides an insight into the presence of areas suitable for Neanderthals outside the already known areas and precisely during the period when a decrease in the size of the Neanderthal population as a whole is expected. Despite this, our research contributes to the large number of studies that are particularly interested in the transition between Neanderthal and modern humans [31–33].

Our study shows that the most important changes in the extent of ecologically suitable areas for Neanderthals occurred between the P1 (90–83 kyr) and P2 (83–69 kyr). This broad result was not unexpected because the second period is warmer than the first. What has not been shown before is that from the second period onwards the "habitability" for Neanderthals in Europe does not change significantly. The European climate simulated in the models we have used here does of course change, becoming generally cooler and drier again after 69 kyr. However, these changes are not geographically uniform and they are not large enough to significantly alter the regions our analysis shows were most suitable for Neanderthal occupation. This remains true when considering the envelope of model uncertainty established by our use of two different climate models. The warming in western, central and southern continental Europe has most effect in expanding the zone of habitability during the period 83–69 kyr. However, the reductions in minimum annual temperature after 69 kyr are centered in the north of our domain, from the eastern coast of the British Isles across Scandinavia into Russia, extending south to northern continental Europe. These are not areas that the period 83–69 kyr warming appear to have made suitable for Neanderthal occupation and the major climate changes in our simulation dataset only impact habitability scores around the margins of that favored area. Although we have shown that temperature is the dominant control of the factors we have analysed, it is not the only one; the period 83–69 kyr sees a general increase in precipitation and reduction in glacial dry conditions, and although this is partly reversed after 69 kyr the spatial pattern is not as uniform as seen for the cooling. Again, the areas with most drying are largely away from the central zone that is favorable for habitability. The climate model simulations we have used for this analysis are of course not perfect and it is possible that they are not reproducing these spatial patterns correctly. However, repeating our analysis using the output of Singarayer simulations [13], which used a different climate model run according to a different protocol with different reconstructions of the northern hemisphere ice sheets, produces broadly similar changes in areas suitable for Neanderthal habitability through our chosen periods. Results from two models support the idea that large-scale climate change in Europe after 69 kyr is not a simple dominating factor in determining the fate of the Neanderthals. Our results appear to be at odds with recent publications [34,35]. Nevertheless, we have shown that climate change, if considered over time (and not as a contrast between extreme periods), has not significantly altered suitable niches for Neanderthals.

If climate change was not the primary driver in the decline of Neanderthal populations in Europe, then what was? During the periods studied here the Neanderthals had a small population size [36–38], were divided into at least three different populations [39], could have suffered a reduction of young female fertility that drove them to extinction [40] and that they may have been susceptible to haemolytic disease of the fetus and newborn (erythroblastosis fetalis), due to maternofetal Rh incompatibility [41].

How can we explain the theoretical contradiction between the decrease of the population size and an increase/stability in the area of suitable territory for Neanderthals? It is possible that the fragmented population found itself restricted to areas that became less suitable (e.g. southern Europe/ Iberia [42]. This may have had a dual effect: preventing movement to more suitable areas of central Europe and simultaneously reducing or even canceling out migration between groups, which is essential to maintain a certain genetic variability. Each of these effects alone may have been responsible for the disappearance of Neanderthals. If the two effects were associated then the probability of Neanderthal survival was almost null.

It should not be forgotten that *Homo sapiens* began to permanently occupy Euro-Asian territory in this same period (also supported by genetic result of interbreeding [43–45]). Although the propositions of direct confrontations between *Homo sapiens* and Neanderthals have not been substantiated, the presence of *Homo sapiens* alone may have had important consequences on Neanderthals ability to occupy the most suitable niches, or simply to continue to exist [46–49].

The presence in the same environment of two or more species with the same characteristics is a problem that has been studied extensively in the field of biology since Gause [50]. The conclusion that two species occupying the same niche in a homogeneous environment cannot coexist and that one excluding the other leads inexorably to the disappearance of one of them has been discussed at length over time through modelling and field experiments for various animal and plant species (for a review see McPeek [51]).

We have evidence that some sites were occupied probably at the same time by *Homo sapiens* and Neanderthal [52], demonstrating that the two species had the same taste in choosing their sites. In addition, Neanderthals had long since begun a demographic decline while *Homo sapiens* could count on influxes of new individuals from Africa and partly from Asia, increasing the indirect effect on the demographic performance of Neanderthals.

## Conclusion

Our research has reconstructed the areas ecologically suitable for Neanderthal occupation in Europe between 90 and 50 kyr BP. We have shown that some of these areas have remained constant, others have changed in extent, and others have disappeared. We compared the evolution of these areas over time, and note that in the most recent periods analyzed, there was not a significant decrease in suitable areas. We therefore propose that environmental factors in Europe as a whole were unlikely to be responsible for predisposing to the disappearance of Neanderthals.

## Supporting information

S1 File Neanderthal fossil sites. (PDF)

S2 File Treatment of the climate data. (PDF)

S3 File Friction and carrying capacity. (PDF)

S4 File ROC analysis. (PDF)

S5 File Variables contribution to the model. (PDF)

S6 File Percentage of ecological niches suitable to Neanderthals. (PDF)
