# Supplementary material for: Climate change in Europe between 90 and 50 kyr BP and Neanderthal territorial habitability
Source: PLoS One. 2025 Feb 26;20(2):e0308690. doi: 10.1371/journal.pone.0308690 (PMC11864554; doi:10.1371/journal.pone.0308690)
Supplement: S1 File — (PDF) [file pone.0308690.s001.pdf]

S1 Neanderthal fossil sites

The list of the sites used in the article (Figure 1). We have taken into consideration sites where fossil remains (or lithic remains) have been attributed to Neanderthals and dated between 90 and 50 Kyrs.

N	Site Name	Reference
1	Goyet Caves	(1)
2	Scladina	(2)
3	La Chaise, abri Bourgois-Delaunay	(3)
4	La Ferrassie	(4)
5	Roc de Marsal	(5)
6	Le Portel	(6)
7	La Rouquette	(7)
8	Grotte de Saint Marcel	(8)
9	Hohlenstein-Stadel	(2,9)
10	Hunas	(10)
11	Buhlen	(11)
12	Sarstedt	(11)
13	Kefalonia	(12)
14	Grotte de kalamakia	(13)
15	Tata	(14)
16	Tabun	(15)
17	Grotta Maggiore di S. Bernardino	(16)
18	Grotta de' Santi	(17)
19	Riparo Mochi	(18)

20	Grotta Madonna dell'Arma	(19)
21	Arma delle Manie	(19)
22	Altai Mountain	(20)
23	Abri Romani	(21)
24	Grotte de Lezetxiki	(22)
25	Valdegoba	(23)
26	El Castillo	(24)
27	Alle-Noir Bois	(25)
28	Korolevo	(26)
29	Staroselye	(27)
30	Kabazi	(28)
31	Prolom II	(29)
32	Teshik-Tash	(27)
33	Kulna	(30)
34	Marillac	(31)
35	Sandougne et abri Brouillard	(32)
36	Grotte de L'Hyene	(33)
37	Regourdou	(34)
38	Grotte d'Unikoté	(35)
39	Grottes d'Isturitz et d'Oxocelhaya	(36)
40	Pech l'Azé1	(37)
41	Abri Laborde	(38)
42	La Brèche de Genay	(39)
43	Grotte de la Verrerie	(40)

44	Loton	(7,41)
45	Préletang	(42)
46	Abri Pié Lombard	(7)
47	Salzgitter-Lebenstedt	(43)
48	Warendorf	(44)
49	Kokkinopilos	(45)
50	Riparo Zampieri	(46)
51	Caverna delle Fate	(47)
52	Tor Faraj	(48)
53	Conceicao	(49)
54	Mezmaiskaya Cave	(50)
55	Chagyrskaya	(51)
56	Divje Babe	(52)
57	Pinilla del Valle	(53)
58	Grotte de Cotencher	(54)
59	Dederiyeh	(55)
60	Molodova	(56)
61	Kiik-Koba	(29)
62	Obi-Rakhamat Grotto	(57)
63	La Quina	(58)
64	Subalyuk	(59)
65	Ein Qashish	(60)
66	Amud	(61)
67	Grotta della Ghiacciaia	(62)

68	Guattari	(63)
69	Grotta di Cotariova	(64)
70	Vilas ruivas	(65)
71	Cueva Anton	(66)
72	Banyoles	(67)
73	Salzofen Cave	(68)
74	Temnata Cave	(69)
75	Fonseigner	(7)
76	Grottes du Coupe-Gorge	(70)
77	Sites du Cotentin	(71)
78	Mauran	(72)
79	Le Moustier	(73)
80	Fieux	(74)
81	La Chapelle aux Saints	(75)
82	Hortus	(76)
83	Grotte Boccard	(77)
84	Königsaue	(78)
85	Shanidar	(79)
86	Kebara	(80)
87	Riparo Tagliente	(81)
88	Grotta Taddeo	(82)
89	Grotta Tina	(83)
90	Bisceglie	(84)
91	Grotta di Uluzzo	(85)

92	Tor Sabiha	(86)
93	Ochoz	(87)
94	El Salt	(88)
95	Gibraltar 2	(89)
96	Cova del Gegant	(90)
97	Cueva del Boquette	(49)
98	Gibraltar 1	(89)
99	Région de la Löwenbourg	(91)
100	Lynford Quarry	(92)
101	Pin Hole Cave	(93)
102	Kryegjata B	(94)
103	Fonds de forêt	(1)
104	Zobiste	(95)
105	Kadar	(95)
106	Roche-Cotard Cave	(96)
107	Grotte de Gatzarria	(97)
108	La Roquette	(7)
109	Combe Grenal	(98)
110	Grottes du Tuteil and de Caougno	(99)
111	Moula Guercy	(100)
112	Auzières 2	(101)
113	Grotte Vaufrey	(102)
114	Calascio	(103)
115	Molare	(82)

116	Northsea shore	(104)
117	Stajnia	(105)
118	San Antao do Tojal	(49)
119	Furninha	(106)
120	Gabasa	(107)
121	El Sidron	(108)
122	Grotte des Plaints	(109)
123	Quneitra	(7)

References

1. Devièse T, Abrams G, Hajdinjak M, Pirson S, De Groote I, Di Modica K, et al. Reevaluating the timing of Neanderthal disappearance in Northwest Europe. Proc Natl Acad Sci USA. 2021 Mar 23;118(12):e2022466118.

2. Peyrégne S, Slon V, Mafessoni F, De Filippo C, Hajdinjak M, Nagel S, et al. Nuclear DNA from two early Neandertals reveals 80,000 years of genetic continuity in Europe. Sci Adv. 2019 Jun;5(6):eaaw5873.

3. Condemi S. Les néandertaliens de LaChaise (abri Bourgeois-Delaunay). Paris: Éd. du Comité des Travaux Historiques et Scientifiques [u.a.]; 2001. 178 p. (Documents préhistoriques).

4. Guérin G, Frouin M, Talamo S, Aldeias V, Bruxelles L, Chiotti L, et al. A multi-method luminescence dating of the Palaeolithic sequence of La Ferrassie based on new excavations adjacent to the La Ferrassie 1 and 2 skeletons. Journal of Archaeological Science. 2015 Jun;58:147–66.

5. Hodgkins J, Marean CW, Turq A, Sandgathe D, McPherron SJP, Dibble H. Climate-mediated shifts in Neandertal subsistence behaviors at Pech de l'Azé IV and Roc de Marsal (Dordogne Valley, France). J Hum Evol. 2016 Jul;96:1–18.

6. Becam G, Chevalier T. Neandertal features of the deciduous and permanent teeth from Portel-Ouest Cave (Ariège, France). American J Phys Anthropol. 2019 Jan;168(1):45–69.

7. Valladas H, Chadelle JP, Geneste JM, Joron JL, Meignen L, Texier PJ. Datations par la thermoluminescence de gisements moustériens du Sud de la France. L'Anthropologie. 1987 Jan;91:211–26.

8. Crégut-Bonnoure E, Faure M, Fernandez P, Guérin C, Moncel MH, Daujeard C. L'occupation de la grotte de Saint-Marcel (Ardèche, France) au Paléolithique moyen : stratégie d'exploitation de l'environnement et type d'occupation de la grotte. L'exemple des couches i, j et j'. Bulletin de la Société préhistorique française. 2004;101(2):257–304.

9. Richard M, Falguères C, Pons-Branchu E, Richter D, Beutelspacher T, Conard NJ, et al. The Middle to Upper Palaeolithic transition in Hohlenstein-Stadel cave (Swabian Jura, Germany): A comparison between ESR, U-series and radiocarbon dating. Quaternary International. 2020 Aug; 556:49–57.

10. Alt KW, Kaulich† B, Reisch L, Vogel H, Rosendahl W. The Neanderthalian molar from Hunas, Germany. HOMO. 2006 Aug;57(3):187–200.

11. Jöris O, Uomini N. Evidence for Neanderthal Hand Preferences from the Late Middle Palaeolithic Site of Buhlen, Germany: Insights into Neanderthal Learning Behaviour. In 2019. p. 77–94.

12. Ferentinos G, Gkioni M, Geraga M, Papatheodorou G. Early seafaring activity in the southern Ionian Islands, Mediterranean Sea. Journal of Archaeological Science. 2012 Jul;39(7):2167–76.

13. Lumley H de, Darlas A. Fouille franco-hellénique de la grotte de Kalamakia (Aréopolis, Péloponnèse). Bulletin de Correspondance Hellénique. 1998;122(2):655–61.

14. Borel A, Dobosi V, Moncel MH. Neanderthal's microlithic tool production and use, the case of Tata (Hungary). Quaternary International. 2017 Apr; 435: 5–20.

15. Jelinek AJ, Farrand WR, Haas G, Horowitz A, Goldberg P. New excavations at the Tabun cave, Mount Carmel, Israel, 1967-1972 : A preliminary report. paleo. 1973;1(2):151–83.

16. Picin A, Peresani M, Falguères C, Gruppioni G, Bahain JJ. San Bernardino Cave (Italy) and the Appearance of Levallois Technology in Europe: Results of a Radiometric and Technological Reassessment. Caramelli D, editor. PLoS ONE. 2013 Oct 16;8(10):e76182.

17. Moroni A, Boschian G, Crezzini J, Montanari-Canini G, Marciani G, Capecchi G, et al. Late Neandertals in central Italy. High-resolution chronicles from Grotta dei Santi (Monte Argentario - Tuscany). Quaternary Science Reviews. 2019 Aug;217:130–51.

18. Douka K, Grimaldi S, Boschian G, Del Lucchese A, Higham TFG. A new chronostratigraphic framework for the Upper Palaeolithic of Riparo Mochi (Italy). Journal of Human Evolution. 2012 Feb;62(2):286–99.

19. Kaniewski D, Renault-Miskovsky J, De Lumley H. Palaeovegetation from a Homo neanderthalensis occupation in Western Liguria: archaeopalynology of Madonna dell'Arma (San Remo, Italy). Journal of Archaeological Science. 2005 Jun;32(6):827–40.

20. Prüfer K, Racimo F, Patterson N, Jay F, Sankararaman S, Sawyer S, et al. The complete genome sequence of a Neanderthal from the Altai Mountains. Nature. 2014 Jan;505(7481):43–9.

21. Sharp WD, Mertz-Kraus R, Vallverdu J, Vaquero M, Burjachs F, Carbonell E, et al. Archeological deposits at Abric Romaní extend to 110 ka: U-series dating of a newly cored, 30 meter-thick section. Journal of Archaeological Science: Reports. 2016 Feb;5:400–6.

22. Garcia-Ibaibarriaga N, Arrizabalaga Á, Iriarte-Chiapusso MJ, Rofes J, Murelaga X. The return to the Iberian Peninsula: first Quaternary record of Muscardinus and a palaeogeographical overview of the genus in Europe. Quaternary Science Reviews. 2015 Jul;119:106–15.

23. Quam RM, Arsuaga JL, Bermúdez De Castro JM, Díez CJ, Lorenzo C, Carretero M, et al. Human remains from Valdegoba Cave (Huérmeces, Burgos, Spain). Journal of Human Evolution. 2001 Nov;41(5):385–435.

24. Rink WJ. Electron spin resonance (ESR) dating and ESR applications in quaternary science and archaeometry. Radiation Measurements. 1997 Dec;27(5–6):975–1025.

25. Hajdas I, Ivy-Ochs S, Pickering R, Preussner F. Recent developments in Quaternary dating methods. Geographica Helvetica. 2008 Sep;63:176–80.

26. Adamenko OM, Gladiline VN. Korolevo - un des plus anciens habitats acheuléens et moustériens de Transcarpatie soviétique. L'Anthropologie. 1990;94:689–712.

27. Pettitt P. The Palaeolithic Origins Of Human Burial. The Palaeolithic Origins of Human Burial. 2010 Dec;1–307.

28. Chabai V, Richter J, Uthmeier T. Kabazi II: Last Interglacial Occupation, Environment & Subsistence. 2006.

29. Stepanchuk V, Sapozhnikov I. The Middle and Upper Pleistocene of Ukraine: A Synopsis of Palaeolithic Finds with Special Reference to Patterns of Peopling and Cultural Development. 2016 [cited 2024 Dec 27]; Available from: http://journals.ub.uni-heidelberg.de/index.php/amold/article/view/29738

30. Michel V, Bocherens H, Valoch K, Yokoyama Y. La grotte de Kulna : analyses physico-chimique et radiométrique des os et dentines de grands mammifères des niveaux du Paléolithique moyen. archeosciences. 2006;30:137–42.

31. Garralda MD, Maureille B, Le Cabec A, Oxilia G, Benazzi S, Skinner MM, et al. The Neanderthal teeth from Marillac (Charente, Southwestern France): Morphology, comparisons and paleobiology. Journal of Human Evolution. 2020 Jan;138:102683.

32. Darpeix A. Nouvelles fouilles à Tabaterie (Dordogne). Gisement Sandougne (Sendonnie). bspf. 1936;33(6):417–41.

33. Enloe JG. Middle Palaeolithic Cave Taphonomy: Discerning Humans from Hyenas at Arcy-sur-Cure, France. Intl J of Osteoarchaeology. 2012 Sep;22(5):591–602.

34. Pelletier M, Royer A, Holliday TW, Discamps E, Madelaine S, Maureille B. Rabbits in the grave! Consequences of bioturbation on the Neandertal "burial" at Regourdou (Montignac-sur-Vézère, Dordogne). Journal of Human Evolution. 2017 Sep;110:1–17.

35. Michel P, Armand D, Couture C, Griggo C, Guadelli JL, Parent G, et al. À propos de la grotte d'Unikoté (Iholdy, Pyrénées-Atlantiques). Topographie, Anthropologie, Palynologie, Paléontologie (Équidés, Hyénidés et Ursidés). Archéologie des Pyrénées Occidentales et des Landes. 1996;15:13–32.

36. Normand C, Cattelain P, editors. La grotte d'Isturitz: fouilles anciennes et récentes: actes de la table ronde du cinquantenaire du classement comme monument historique des grottes d'Isturitz et d'Oxocelhaya sous la direction de Christian Normand, Hasparren, 14-15 novembre 2003. Treignes: Cedarc; 2017. 244 p. (Artéfacts).

37. Rendu W. Hunting behavior and Neanderthal adaptability in the Late Pleistocene site of Pech-de-l'Azé I. Journal of Archaeological Science. 2010 Aug;37(8):1798–810.

38. Vaissié E, Delvigne V, Faivre JP, Fernandes P, Turq A, Raynal JP. Techno-économie et signification culturelle de l'occupation moustérienne supérieure de Baume-Vallée (Haute-Loire). Comptes Rendus Palevol. 2017 Sep;16(7):804–19.

39. Garralda MD, Maureille B, Pautrat Y, Vandermeersch B. La molaire d'enfant néandertalien de Genay (Côte-d'Or, France). Réflexions sur la variabilité dentaire des Néandertaliens. paleo. 2008 Dec 1;(20):89–100.

40. Piveteau J. Traité de paléontologie. Vol. 7. Paris: Masson & Cie; 1957. 670 p.

41. Geneste JM, Jaubert J, Lenoir M, Meignen L, Turq A. Approche technologique des Moustériens Charentiens du Sud-Ouest de la France et du Languedoc oriental. pal. 1997;9(1):101–42.

42. Griggo C, Bernard-Guelle S, Tillet T, Argant A. La grotte de Prélétang (Presles, Isère) : un camp de chasse moustérien de moyenne montagne. edyte. 2018;20(1):179–94.

43. Ruebens K, Smith GM, Fewlass H, Sinet-Mathiot V, Hublin J, Welker F. Neanderthal subsistence, taphonomy and chronology at Salzgitter-Lebenstedt (Germany): a multifaceted analysis of morphologically unidentifiable bone. J Quaternary Science. 2023 May;38(4):471–87.

44. Scholz M, Czarnetzki A, Pusch C. New Evidence of Neanderthal Man in Northern Germany: A Fragment of an Os parietale from Late Pleistocene Sediments at Warendorf-Neuwarendorf. Bull Soc Suisse d`Anthrop. 2000 Jan;6:1–7.

45. Tourloukis V, Karkanas P, Wallinga J. Revisiting Kokkinopilos: Middle Pleistocene radiometric dates for stratified archaeological remains in Greece. Journal of Archaeological Science. 2015 May;57:355–69.

46. Palma Di Cesnola A. Gli scavi nel Riparo Zampieri presso Verona. In: Memorie del Museo Civico di Storia Naturale. Verona; 1961. p. 273–90.

47. De Lumley MA, Giacobini G. Les néandertaliens de la Caverna delle Fate (Finale Ligure, Italie). I - Chronostratigraphie, restes squelettiques. L'Anthropologie. 2013 Jun;117(3):273–304.

48. Groucutt HS. Middle Palaeolithic point technology, with a focus on the site of Tor Faraj (Jordan, MIS 3). Quaternary International. 2014 Nov;350:205–26.

49. Zilhão J. Chronostratigraphy of the Middle-to- Upper Paleolithic Transition in the Iberian Peninsula. Pyrenae. 2006 Jan;37(1):7–84.

50. Skinner AR, Blackwell BAB, Martin S, Ortega A, Blickstein JIB, Golovanova LV, et al. ESR dating at Mezmaiskaya Cave, Russia. Applied Radiation and Isotopes. 2005 Feb;62(2):219–24.

51. Mafessoni F, Grote S, De Filippo C, Slon V, Kolobova KA, Viola B, et al. A high-coverage Neandertal genome from Chagyrskaya Cave. Proc Natl Acad Sci USA. 2020 Jun 30;117(26):15132–6.

52. Turk M, Turk J. On the Significance of Divje babe I Cave for the Stratigraphy, Sedimentology, and Chronology of Palaeolithic Cave Sites in Slovenia. AV. 2023 Jul 14;74:7–38.

53. Álvarez-Alonso D, De Andrés-Herrero M, Díez-Herrero A, Medialdea A, Rojo-Hernández J. Neanderthal settlement in central Iberia: Geo-archaeological research in the Abrigo del Molino site, MIS 3 (Segovia, Iberian Peninsula). Quaternary International. 2018 Apr;474:85–97.

54. Nielsen E. Response of the Lateglacial fauna to climatic change. Palaeogeography, Palaeoclimatology, Palaeoecology. 2013 Dec;391:99–110.

55. Akazawa T, Muhesen S, Ishida H, Kondo O, Griggo C. New Discovery of a Neanderthal Child Burial from the Dederiyeh Cave in Syria. paleo. 1999;25(2):129–42.

56. Haesaerts P, Damblon F, Nigst P, Hublin JJ. ABA and ABOx Radiocarbon Cross-Dating on Charcoal from Middle Pleniglacial Loess Deposits in Austria, Moravia, and Western Ukraine. Radiocarbon. 2013;55(2):641–7.

57. Skinner AR, Blackwell BAB, Mian A, Baboumian SM, Blickstein JIB, Wrinn PJ, et al. ESR analyses on tooth enamel from the Paleolithic layers at the Obi-Rakhmat hominid site, Uzbekistan: Tackling a dating controversy. Radiation Measurements. 2007 Jul;42(6–7):1237–42.

58. Debénath A, Dibble HL. Nouvelles fouilles à La Quina (Charente) : résultats préliminaires. galip. 1998;40(1):31–3.

59. Mester Z, Coqueugniot H, Tillier AM, Rosendahl W, Friedrich R, Zink A, et al. First direct dating of the Late Neanderthal remains from Subalyuk Cave in Northern Hungary. anthranz. 2024 Mar 21;81(2):169–81.

60. Been E, Hovers E, Ekshtain R, Malinski-Buller A, Agha N, Barash A, et al. The first Neanderthal remains from an open-air Middle Palaeolithic site in the Levant. Sci Rep. 2017 Jun 7;7(1):2958.

61. Rink WJ, Schwarcz HP, Lee HK, Rees-Jones J, Rabinovich R, Hovers E. Electron spin resonance (ESR) and thermal ionization mass spectrometric (TIMS)^{230} Th/^{234} U dating of teeth in Middle Paleolithic layers at Amud Cave, Israel. Geoarchaeology. 2001 Aug;16(6):701–17.

62. Peresani M. Inspecting human evolution from a cave. Late Neanderthals and early sapiens at Grotta di Fumane: present state and outlook. Journal of Anthropological Sciences. 2022;(100):71–107.

63. Grün R, Stringer CB. ELECTRON SPIN RESONANCE DATING AND THE EVOLUTION OF MODERN HUMANS. Archaeometry. 1991 Aug;33(2):153–99.

64. Andreolotti S, Gerdol R. L'industria mousteriana della grotta Cotariova (carso Triestino). Atti et Mem della Commissione Grotte « Eugenio Boegan ». 1974;XIII:113–31.

65. Paixão E, Caninas J, Carvalho E, Figueiredo O, Henriques F, Maio D, et al. The Mousterian site of Cobrinhos, Vila Velha de Ródão (Portugal). In: Sociedade dos Amigos do Museu de Francisco Tavares Proença Júnior. 2016. p. 135–50.

66. Zilhão J. The late persistence of the Middle Palaeolithic and Neandertals in Iberia: A review of the evidence for and against the "Ebro Frontier" model. Quaternary Science Reviews. 2021 Oct;270:107098.

67. Grün R, Maroto J, Eggins S, Stringer C, Robertson S, Taylor L, et al. ESR and U-series analyses of enamel and dentine fragments of the Banyoles mandible. Journal of Human Evolution. 2006 Mar;50(3):347–58.

68. Kipfer BA. Encyclopedic Dictionary of Archaeology [Internet]. Cham: Springer International Publishing; 2021 [cited 2024 Dec 24]. Available from: https://link.springer.com/10.1007/978-3-030-58292-0

69. Ruiz-Redondo A, Davies W. The Prehistoric Hunter-Gatherers of South-Eastern Europe. 2023.

70. Martínez De Pinillos M, Martín-Francés L, De Castro JMB, García-Campos C, Modesto-Mata M, Martinón-Torres M, et al. Inner morphological and metric characterization of the molar remains from the Montmaurin-La Niche mandible: The Neanderthal signal. Journal of Human Evolution. 2020 Aug;145:102739.

71. Cliquet D, Mercier N, Valladas H, Froget L, Michel D, Vliet-Lanoë BV, et al. Apport de la thermoluminescence sur silex chauffés à la chronologie de sites paléolithiques de Normandie : nouvelles données et interprétations. Quaternaire. 2003;14(1):51–64.

72. Valladas H, Mercier N, Falguères C, Bahain JJ. Contribution des méthodes nucléaires à la chronologie des cultures paléolithiques entre 300 000 et 35 000 ans BP. galip. 1999;41(1):153–66.

73. Higham T, Douka K, Wood R, Ramsey CB, Brock F, Basell L, et al. The timing and spatiotemporal patterning of Neanderthal disappearance. Nature. 2014 Aug 21;512(7514):306–9.

74. Laroulandie V, Faivre JP, Gerbe M, Mourre V. Who brought the bird remains to the Middle Palaeolithic site of Les Fieux (Southwestern, France)? Direct evidence of a complex taphonomic story. Quaternary International. 2016 Nov;421:116–33.

75. Rendu W, Beauval C, Crevecoeur I, Bayle P, Balzeau A, Bismuth T, et al. Evidence supporting an intentional Neandertal burial at La Chapelle-aux-Saints. Proc Natl Acad Sci USA. 2014 Jan 7;111(1):81–6.

76. de Lumley H. La grotte de l'Hortus. Les chasseurs néandertaliens et leur milieu de vie. Elaboration d'une chronologie du Wurmien II dans le Midi méditerranéen. 1972. 656 p. (Etudes quaternaires; vol. 1).

77. Maureille B, Djindjian F, Garralda MD, Mann AE, Vandermeersch B. Les dents moustériennes de la grotte Boccard, lieu-dit Bas-de-Morant (commune de Créancey, Côte-d'Or, Bourgogne): The Mousterian teeth from the Boccard Cave, Bas-de-Morant (Municipality of Créancey, Côte-d'Or, Bourgogne). bmsap [Internet]. 2008 Jun 1 [cited 2024 Dec 26];20(1–2). Available from: http://journals.openedition.org/bmsap/6047

78. Jöris O. Bifacially backed knives (Keilmesser) in the Central European Middle Palaeolithic. Axe Age: Acheulian Tool-making from Quarry to Discard. 2006 Jan;287–310.

79. Pomeroy E, Mirazón Lahr M, Crivellaro F, Farr L, Reynolds T, Hunt CO, et al. Newly discovered Neanderthal remains from Shanidar Cave, Iraqi Kurdistan, and their attribution to Shanidar 5. Journal of Human Evolution. 2017 Oct;111:102–18.

80. Rebollo NR, Weiner S, Brock F, Meignen L, Goldberg P, Belfer-Cohen A, et al. New radiocarbon dating of the transition from the Middle to the Upper Paleolithic in Kebara Cave, Israel. Journal of Archaeological Science. 2011 Sep;38(9):2424–33.

81. Arnaud J, Peretto C, Panetta D, Tripodi M, Fontana F, Arzarello M, et al. A reexamination of the Middle Paleolithic human remains from Riparo Tagliente, Italy. Quaternary International. 2016 Dec;425:437–44.

82. Benazzi S, Douka K, Fornai C, Bauer CC, Kullmer O, Svoboda J, et al. Early dispersal of modern humans in Europe and implications for Neanderthal behaviour. Nature. 2011 Nov 24;479(7374):525–8.

83. Martini F, Sala B, Bartolomei G, Tonon M, Cattani L. La Grotta Tina a Marina di Camerota (Salerno). Bullettino di Paletnologia Italiana Roma. 1972;81:27–79.

84. Mallegni F, Piperno M, Segre A. Human remains of *Homo sapiens neanderthalensis* from the pleistocene deposit of Sants Croce Cave, Bisceglie (Apulia), Italy. American J Phys Anthropol. 1987 Apr;72(4):421–9.

85. Spinapolice EE, Zerboni A, Meyer MC, Talamo S, Mariani GS, Gliganic LA, et al. Back to Uluzzo – archaeological, palaeoenvironmental and chronological context of the Mid–Upper Palaeolithic sequence at Uluzzo C Rock Shelter (Apulia, southern Italy). J Quaternary Science. 2022 Feb;37(2):217–34.

86. Emery-Barbier A. Analyses polliniques du Quaternaire supérieur en Jordanie méridionale. paleo. 1988;14(1):111–8.

87. Neruda P, Valoch K. Palaeolithic people and Moravian Caves. Univ Masaryk Brun Geology Brno. 2007 Jan;35.

88. Garralda MD, Galván B, Hernández CM, Mallol C, Gómez JA, Maureille B. Neanderthals from El Salt (Alcoy, Spain) in the context of the latest Middle Palaeolithic populations from the southeast of the Iberian Peninsula. J Hum Evol. 2014 Oct;75:1–15.

89. Devièse T, Karavanić I, Comeskey D, Kubiak C, Korlević P, Hajdinjak M, et al. Direct dating of Neanderthal remains from the site of Vindija Cave and implications for the Middle to Upper Paleolithic transition. Proc Natl Acad Sci USA. 2017 Oct 3;114(40):10606–11.

90. Daura J, Sanz M, Pike AWG, Subirà ME, Fornós JJ, Fullola JM, et al. Stratigraphic context and direct dating of the Neandertal mandible from Cova del Gegant (Sitges, Barcelona). Journal of Human Evolution. 2010 Jul;59(1):109–22.

91. Jagher R, Jagher E. Les gisements paléolithiques de la Löwenburg, commune de Pleigne. Archaologie der Schweiz. 1987 Jun;10:43–52.

92. O'Connor S, Edwards HGM, Ali E. An Interim Investigation of the Potential of Vibrational Spectroscopy for the Dating of Cultural Objects in Ivory. archeosciences. 2011;35:159–65.

93. White MJ, Pettitt PB. The British Late Middle Palaeolithic: An Interpretative Synthesis of Neanderthal Occupation at the Northwestern Edge of the Pleistocene World. J World Prehist. 2011 May;24(1):25–97.

94. Runnels C, Korkuti M, Galaty ML, Timpson ME, Whittaker JC, Stocker SR, et al. The Palaeolithic and Mesolithic of Albania: Survey and Excavation at the Site of Kryegjata B (Fier District). Journal of Mediterranean Archaeology. 2004 Jun;17(1):3–29.

95. Montet-White A. Le paléolithique en ancienne Yougoslavie. Grenoble: J. Millon; 1996. (L'homme des origines).

96. Marquet JC, Lorblanchet M, Egels Y, Esquerre-Pourtère J, Hesse MS. Symbolic character productions in the mousterian site of La Roche-Cotard in Langeais (Indre-et-Loire, France) and their geological context. paleo. 2014 Dec 28;(25):169–94.

97. Barshay-Szmidt CC, Eizenberg L, Deschamps M. Radiocarbon (AMS) dating the Classic Aurignacian, Proto-Aurignacian and Vasconian Mousterian at Gatzarria Cave (Pyrénées-Atlantiques, France). paleo. 2012 Dec 15;(23):11–38.

98. Garralda MD, Vandermeersch B. Les Néandertaliens de la grotte de Combe-Grenal (Domme, Dordogne, France) / The Neanderthals from Combe-Grenal cave (Domme, Dordogne, France). pal. 2000;12(1):213–59.

99. Breuil H. Le Moustérien dans l'Ariège. In Orléans : Impr. Paul Pigelet et fils et Cie; 1922. p. 508–11.

100. Hlusko LJ, Carlson JP, Guatelli-Steinberg D, Krueger KL, Mersey B, Ungar PS, et al. Neanderthal teeth from moula-guercy, Ardèche, France. American J Phys Anthropol. 2013 Jul;151(3):477–91.

101. Marchal F, Monchot H, Coussot C, Desclaux E, Deschamp P, Thiébaut C, et al. Neandertals paleoenvironment in Western Provence: The contribution of Les Auzières 2 (Méthamis, Vaucluse, France). Comptes Rendus Palevol. 2009 Jul;8(5):493–502.

102. Garralda MD, Maureille B, Rigaud JP, Vandermeersch B. La molaire néandertalienne de la grotte Vaufrey (Dordogne, France). bmsap [Internet]. 2004 Dec 1 [cited 2024 Dec 24];16(3–4). Available from: http://journals.openedition.org/bmsap/4023

103. Nicoud E, Aureli D, Pagli M. Comportements techniques au Pléistocène moyen en Italie: Nouvelles recherches sur l'industrie lithique et le site de Valle Giumentina (Abruzzes). mefra [Internet]. 2015 Apr 29 [cited 2024 Dec 26];(127–1). Available from: http://journals.openedition.org/mefra/2659

104. White MJ. Things to do in Doggerland when you're dead: surviving OIS3 at the northwestern-most fringe of Middle Palaeolithic Europe. World Archaeology. 2006 Dec;38(4):547–75.

105. Dąbrowski P, Nowaczewska W, Stringer CB, Compton T, Kruszyński R, Nadachowski A, et al. A Neanderthal lower molar from Stajnia Cave, Poland. HOMO. 2013 Apr;64(2):89–103.

106. Cardoso J, Bicho N. Paleolithic occupations and Lithic Assemblages from Furninha Cave, Peniche (Portugal). Zephyrus. 2010 Nov;LXVI, ISSN: 0514-7336:17–38.

107. Lorenzo C, Navazo M, Díez JC, Sesé C, Arceredillo D, Jordá Pardo JF. New human fossil to the last Neanderthals in central Spain (Jarama VI, Valdesotos, Guadalajara, Spain). Journal of Human Evolution. 2012 Jun;62(6):720–5.

108. De Torres T, Ortiz JE, Grün R, Eggins S, Valladas H, Mercier N, et al. Dating of the hominid (*homo neanderthalensis*) remains accumulation from El Sidrón cave (Piloña, Asturias, north Spain): an example of a multi-methodological approach to the dating of upper pleistocene sites. Archaeometry. 2010 Aug;52(4):680–705.

109. Chauvière FX, Blant D, Boudadi-Maligne M, Brenet F, Castel JC, Judit D, et al. La grotte des Plaints. Dans les pas de Jean-Pierre Jéquier (1937-1967) ou retour à la grotte des Plaints (Couvet, NE). Cavernes. 2022 Jun;2021:4–13.
