# Supplementary material for: Climate change in Europe between 90 and 50 kyr BP and Neanderthal territorial habitability
Source: PLoS One. 2025 Feb 26;20(2):e0308690. doi: 10.1371/journal.pone.0308690 (PMC11864554; doi:10.1371/journal.pone.0308690)
Supplement: S2 File — (PDF) [file pone.0308690.s002.pdf]

S2 Treatment of the climate data

The evolution of the climate and biomes during our study period is derived from simulations of the climate of the last glacial cycle, primarily those conducted by (1), which are processed closely following the method of (2). An outline of this procedure will be given here, although for full details we refer interested readers to (2).

The processing has two steps. The climate simulation output is first anomalised to remove known systematic biases in the model and downscale to a higher spatial resolution, and then biomes corresponding to the local climatic conditions are calculated using the BIOME4 environmental model (3).

In the first step, a climatology of monthly anomalies of the surface air temperature, precipitation and cloud cover (a proxy for direct solar insolation) with respect to the model's modern climate are calculated for each of the time periods in our study. These anomalies are then added to monthly climatological maps of observations of those quantities to create the actual climate fields used. For this study, the final climate fields are produced on the 0.5x0.5 degree longitude-latitude grid of the (4) observational climatology provided with the BIOME4 distribution. This effectively downscales the climate fields at the same time as anomalising them, including implicit adjustments to topographic detail present in the real world that are not resolved by the original climate model.

To derive the biomes implied by the climates present in each of the time periods in our study, these climate fields were then used as inputs to the BIOME4 model, along with the contemporaneous atmospheric CO_2 concentration. Soil properties used in this calculation were held constant at modern values for all time periods. As noted in the text, where changes in sea level imply the exposure of coastal grid points in paleo time periods that are not present in the modern datasets used, input variables for BIOME4 were created by extrapolating from the nearest three existing valid grid cells.

The biomes thus calculated are then used to derive maps of human friction and carrying capacity to use as input to Maxent along with the surface temperature and precipitation, using the look-up table shown in Appendix S4.

References

1. Smith RS, Gregory J. The last glacial cycle: transient simulations with an AOGCM. Clim Dyn. 2012 Apr;38(7–8):1545–59.

2. Hoogakker BAA, Smith RS, Singarayer JS, Marchant R, Prentice IC, Allen JRM, et al. Terrestrial biosphere changes over the last 120\,kyr. Climate of the Past. 2016;12(1):51–73.

3. Kaplan JO, Bigelow NH, Prentice IC, Harrison SP, Bartlein PJ, Christensen TR, et al. Climate change and Arctic ecosystems: 2. Modeling, paleodata-model comparisons, and future projections. J Geophys Res. 2003 Oct 16;108(D19):2002JD002559.

4. Leemans R, Cramer W. The IIASA database for mean monthly values of temperature, precipitation, and cloudiness on a global terrestrial grid. International Institut for Applied Systems Analysis (IIASA). 1991;RR-91-18:61.
