# Supplementary material for: Climate change in Europe between 90 and 50 kyr BP and Neanderthal territorial habitability
Source: PLoS One. 2025 Feb 26;20(2):e0308690. doi: 10.1371/journal.pone.0308690 (PMC11864554; doi:10.1371/journal.pone.0308690)
Supplement: S3 File — (PDF) [file pone.0308690.s003.pdf]

S3 Friction and carrying capacity

Friction is defined as the difficulty of moving in a given environment, based primarily on the density of vegetation affecting human movement: the lower the friction of the environment, the easier it is to move around. The friction values used, for each type of global vegetation or biome, were proposed by (1).

Carrying capacity corresponds to the maximum number of individuals who can be supported in a given area within natural resource limits. It is an indicator for the available quantity of flora and fauna. Carrying capacity values were attributed to each type of vegetation using the correspondence table published by (1) and expressed in individuals /10'000 km2

Carrying capacity	Friction	Binford biome categories (2)
2866	0.9	Upper equatorial savanna-woodland/broadleaf free savanna Equatorial and tropical rain forests
2780	0.9	Monsoon (raingreen) forest
892	0.5	Upper tropical thorn forest and thorn woodland Upper subtropical sclerophyllous scrub-dwarf forest
463	0.5	Lower tropical thorntree-desert grass savanna
1451	0.1	Subtropical semidesert scrub Desert alternating with porcupine grass semidesert Midlatitude semidesert scrub and woodland
3196	0.1	Midlatitude short grass prairie
50	0.9	Desert
2209	0.1	Upper subtropical Australian sclerophyllous tree savanna Tropical thorntree-tallgrass savanna
2848	0.5	Lower midlatitude Australian sclerophyll scrub forest Subtropical broadleaf evergreen forest Coastal forest Lake forest Southern pine forest
3429	0.5	Tropical montane forest
2848	0.5	Midlatitude Mediterranean evergreen mixed forest
1002	0.1	Mixed boreal and deciduous forest, Boreal forest, Boreal forest dominated by deciduous larch-aspen, Open boreal woodlands
1345	0.5	Temperate deciduous broad leaved forest Midlatitude deciduous forest
281	0.1	Polar and alpine desert
99	0.1	Moderately dry boreal parkland
885	0.1	Tall grass prairie-forest steppe

References

1 Ray N. Modélisation de la démographie des populations humaines préhistoriques à l'aide de données environnementales et génétiques [doctorat]. [Genève]: Genève; 2003.

2 Binford LR. Constructing frames of reference: an analytical method for archaeological theory building using hunter-gatherer and environmental data sets. Berkeley: University of California Press; 2001. 563 p.
