# Supplementary figures and images for: Climate change in Europe between 90 and 50 kyr BP and Neanderthal territorial habitability
Source: PLoS One. 2025 Feb 26;20(2):e0308690. doi: 10.1371/journal.pone.0308690 (PMC11864554; doi:10.1371/journal.pone.0308690)

## S4 ROC analysis

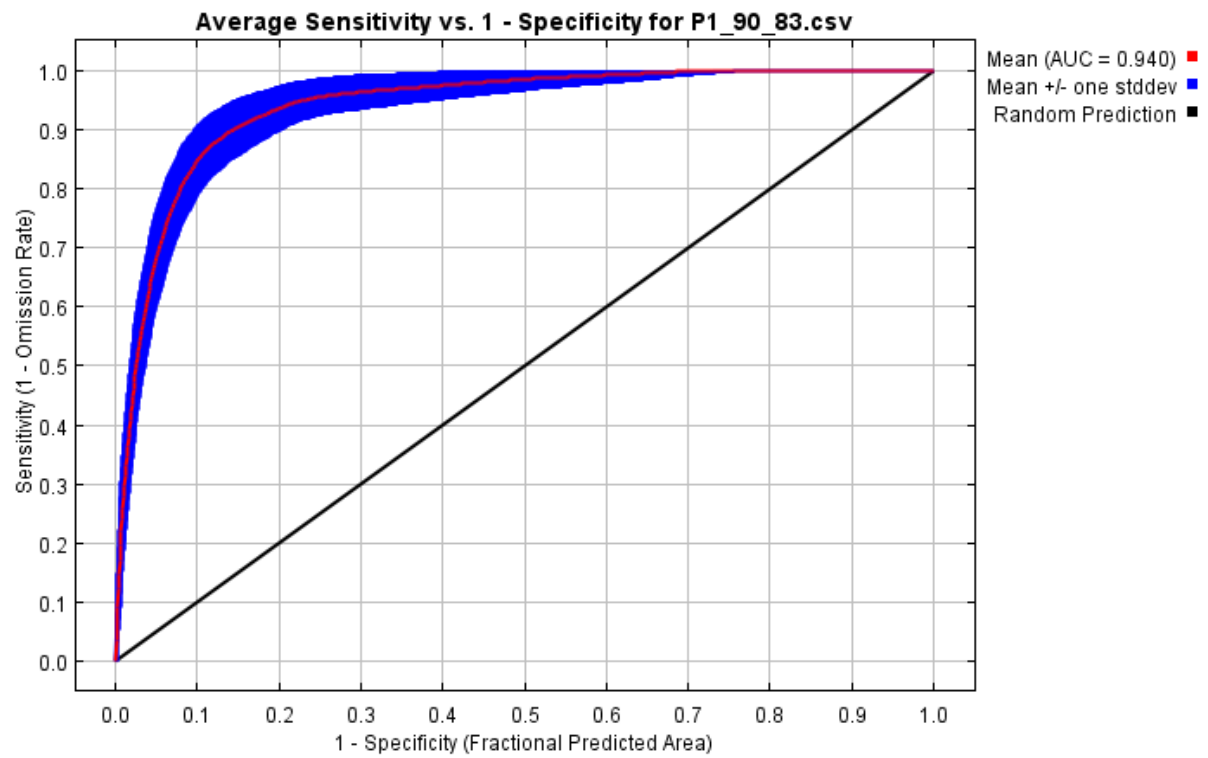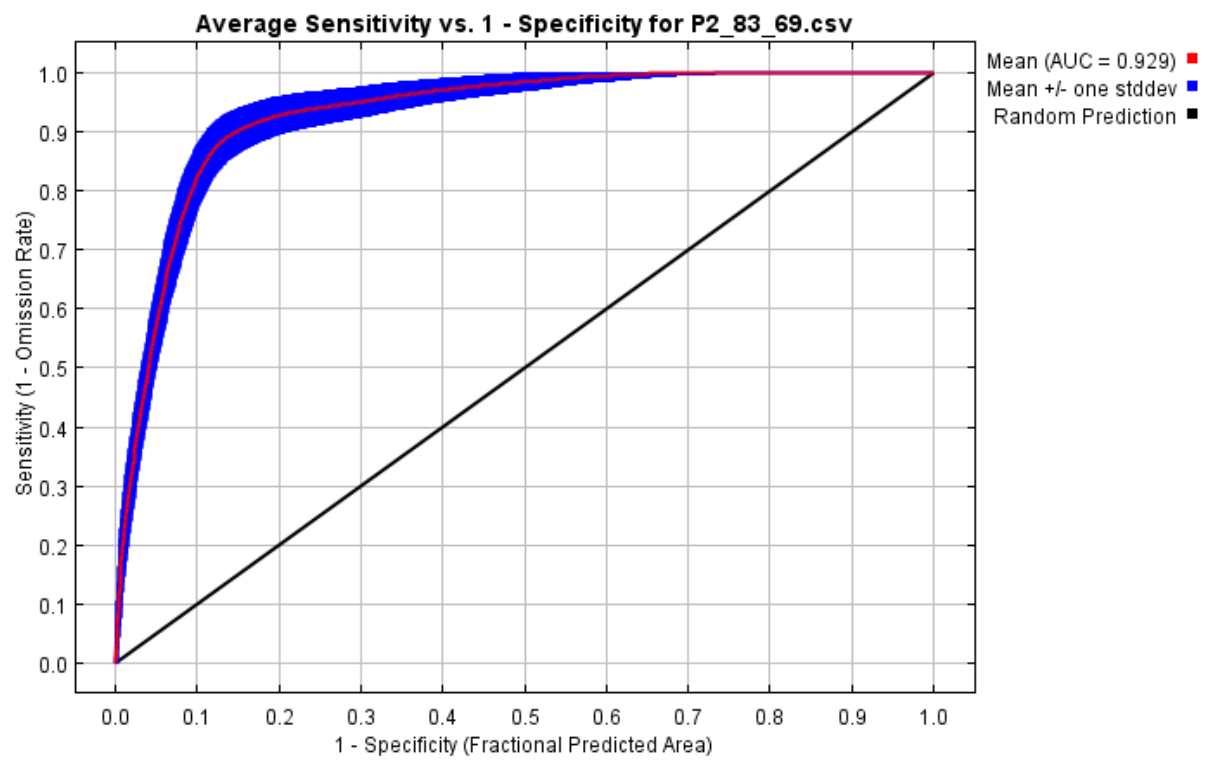

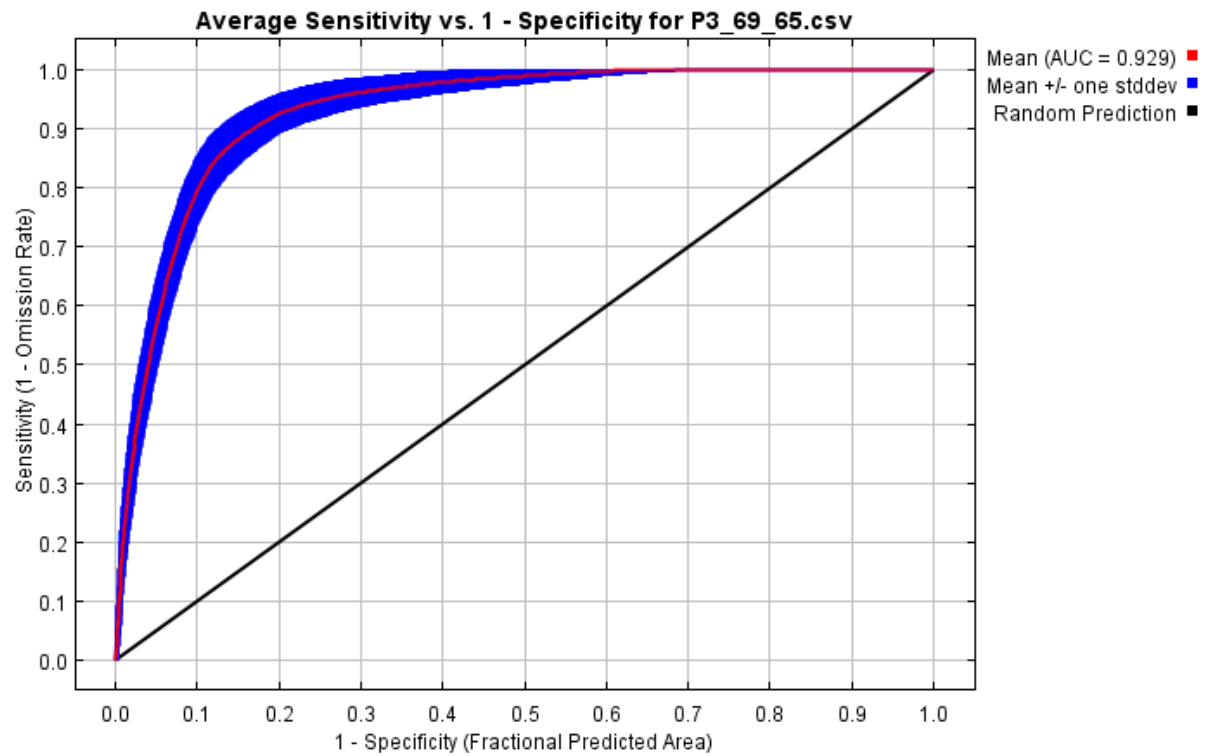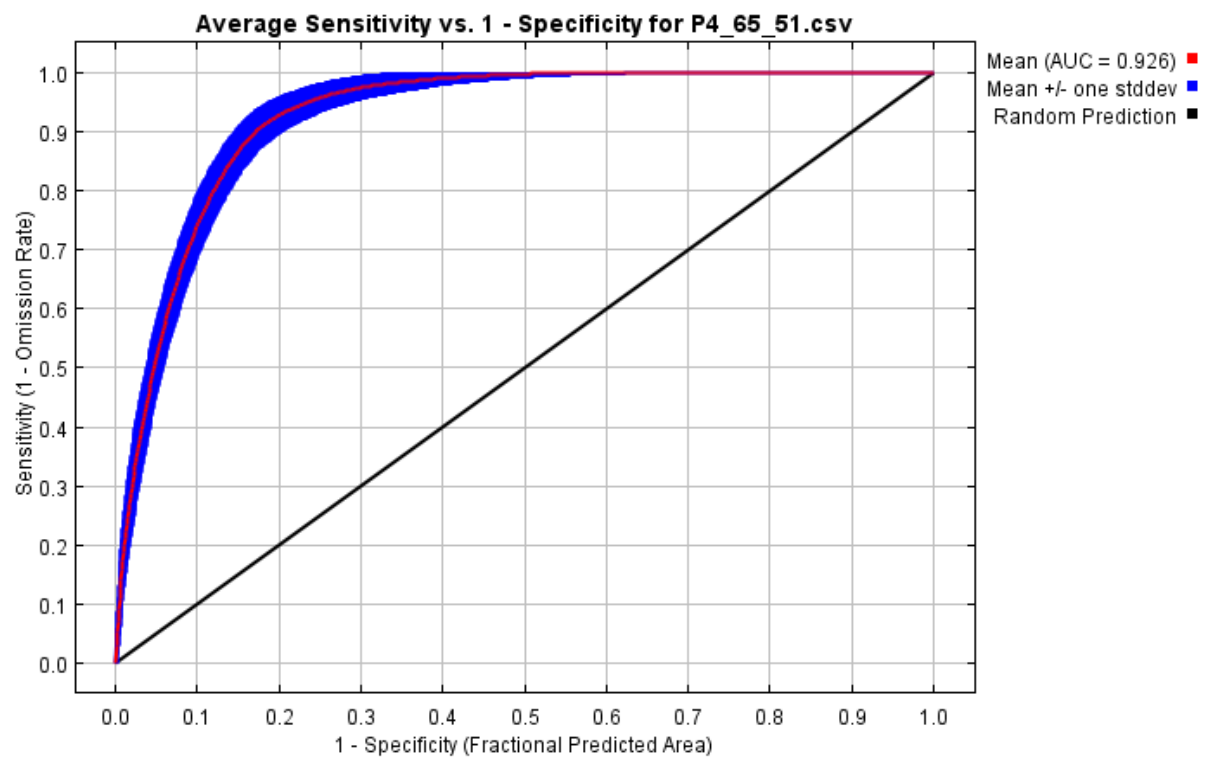

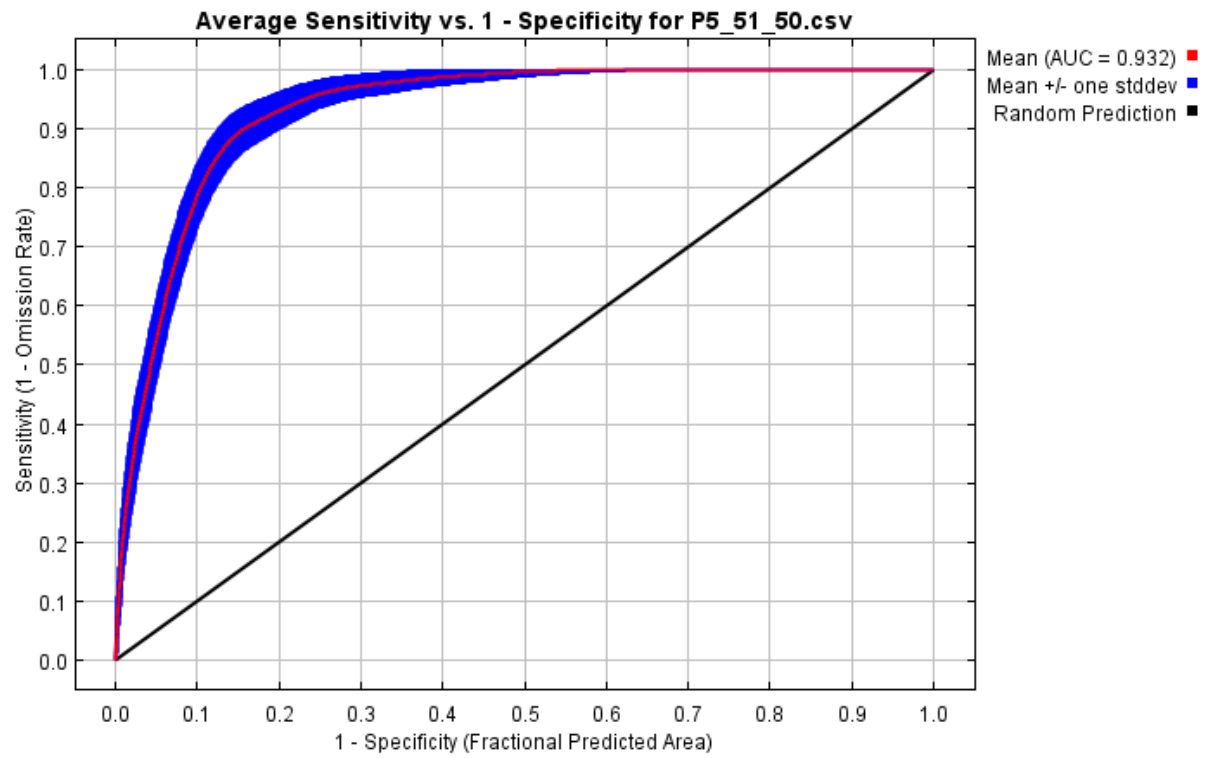

Supplement: S4 File — (PDF) [file pone.0308690.s004.pdf]

## S5 Variables contribution to the model

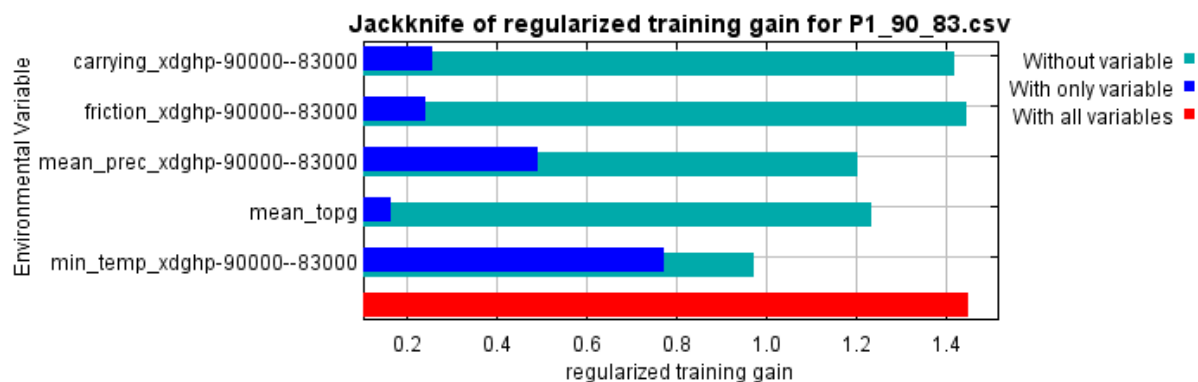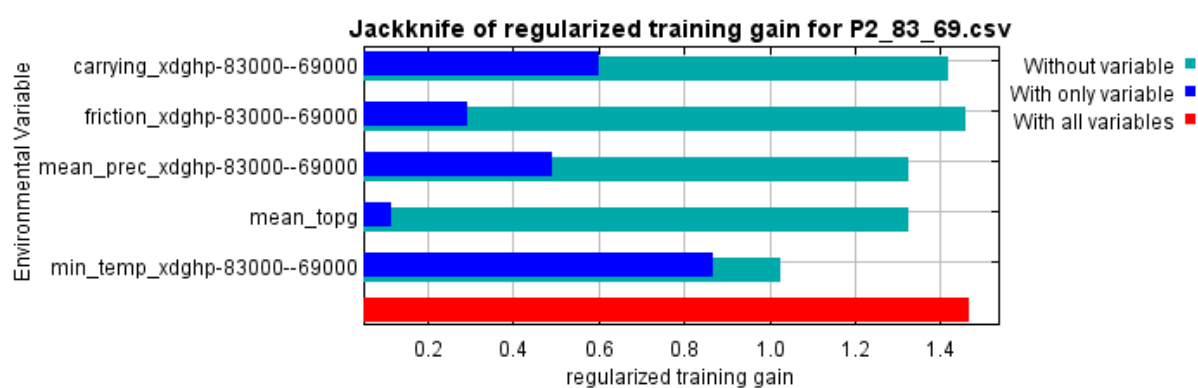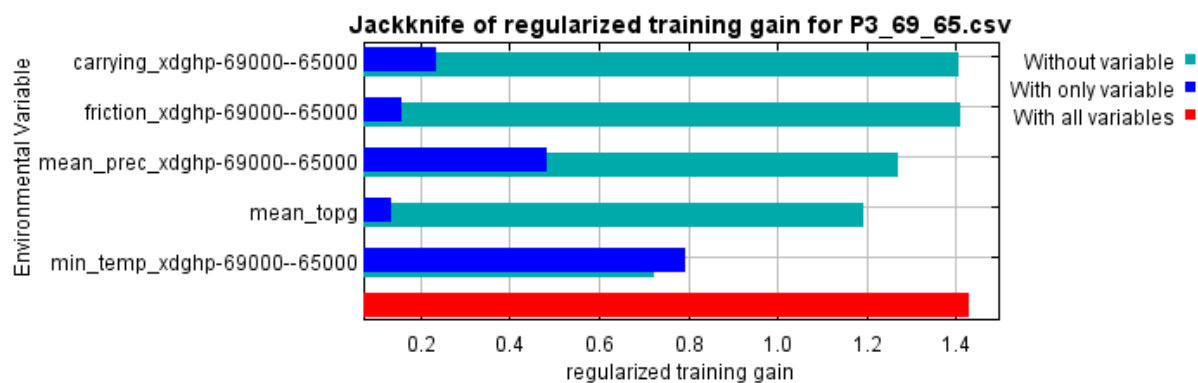

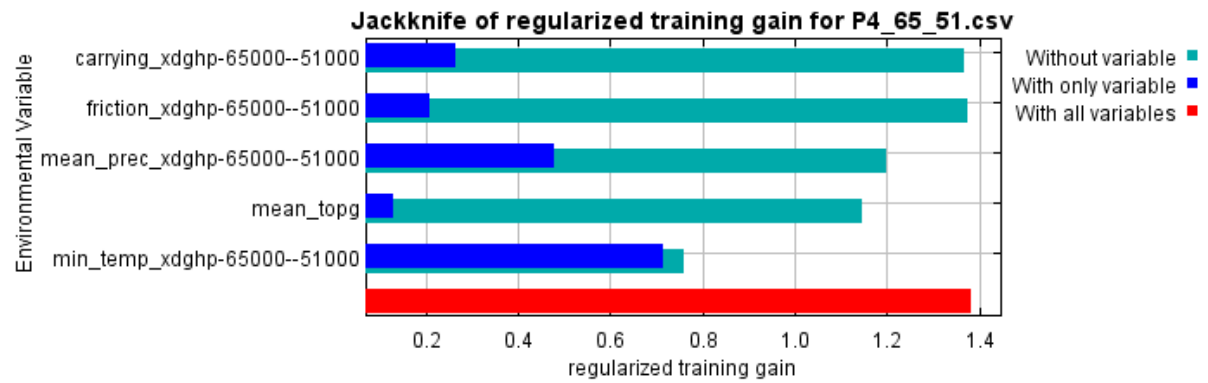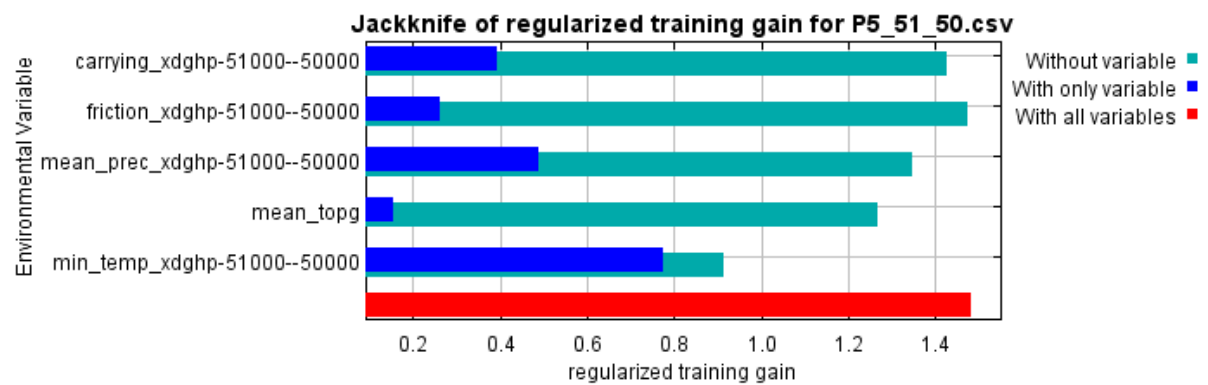

Supplement: S5 File — (PDF) [file pone.0308690.s005.pdf]
