# Supplementary material for: Climate change in Europe between 90 and 50 kyr BP and Neanderthal territorial habitability
Source: PLoS One. 2025 Feb 26;20(2):e0308690. doi: 10.1371/journal.pone.0308690 (PMC11864554; doi:10.1371/journal.pone.0308690)
Supplement: S6 File — (PDF) [file pone.0308690.s006.pdf]

S6 Percentage of ecological niches suitable to Neanderthals

Period	% of pixel having a probability greater than 0.8	% of pixel having a probability greater than 0.9
P1	2.2	0.4
P2	3.3	1.6
P3	3.2	1.0
P4	3.5	1.1
P5	3.1	1.1

Total number of pixels = 21600
